# Understanding determinants of COVID-19 vaccine hesitancy; an emphasis on the role of religious affiliation and individual’s reliance on traditional remedy

**DOI:** 10.1186/s12889-022-13485-2

**Published:** 2022-06-07

**Authors:** Hanna Defar Hassen, Mengistu Welde, Mesay Moges Menebo

**Affiliations:** 1 School of Medicine, Faculty of Medical Sciences, Institute of Health, Jimma University, Jimma, Ethiopia; 2 Department of Biomedical Sciences, Faculty of Medical Sciences, Institute of Health, Jimma University, Jimma, Ethiopia; 3 Department of Business and IT, School of Business, University of South-Eastern Norway, Campus Bø, Notodden, Norway

**Keywords:** COVID-19, Vaccine hesitancy

## Abstract

**Background:**

The damage COVID-19 has caused interms of mortalities, economic breakdown and social disruption is immense. The COVID-19 vaccine has been one of the efficient prevention strategies so far in preventing the pandemic. However, the publics’ hesitancy towards vaccines has enormously affected this task. With emerging research findings indicating that a substantial proportion of adults are hesitant about a vaccine for COVID-19, important work that identifies and describes vaccine hesitant individuals is required to begin to understand and address this problem.

**Objective:**

This study assessed public attitude towards COVID-19 Vaccine and identified important factors that lead to its hesitancy.

**Methods:**

A web and paper-based cross-sectional survey study was conducted from July 31 to August 12, 2021. The study participants are staffs and students at Jimma University. A total of 358 participants were selected using stratified simple random sampling and requested to fill a survey questionnaire. Binomial logistic regression analysis was done to identify factors associated with COVID-19 vaccine hesitancy.

**Results:**

Half of the participants were found to be hesitant to COVID-19 vaccine. The odds of becoming vaccine hesitant among middle income was two times more than those with lower income (AOR 2.17, 95% CI 1.05–4.5). Furthermore, respondent’s extent of exposure was associated with vaccine hesitancy with the odds of becoming vaccine hesitant among those whose source of COVID-19 information is from four media sources (Social Media, Mass Media, Health care worker and Friends/family/Neighbor) being 74% lower (AOR .26, 95% CI .09–.69) than those with one media source. Concern towards vaccine side effect, vaccine effectiveness and having the belief to treat COVID-19 with traditional remedies were found to increase the odds of becoming vaccine hesitant by 31%, 42% and 37% respectively. Moreover, the association between side-effect concern and vaccine hesitancy was moderated by participant’s religious affiliation*.*

**Supplementary Information:**

The online version contains supplementary material available at 10.1186/s12889-022-13485-2.

## Introduction

The global and national damage COVID-19 has caused interms of mortalities, economic breakdown and social disruption is immense. Until this day, it left 3.9 million people dead [1], almost 25% of COVID-19 patients with long term symptoms and organ damages, and 40,000 children parentless [2]. Economic wise, it became a reason for export activities to be plummeted by 46% [3], and 43% of businesses worldwide to be closed [4] bringing significant amount of employees to an economic crisis. With people’s movement impeded, It has also caused experiences of depression and anxiety to surge by 25% [5], and major social activities like the Olympic to be postponed.

So far, there are 19 COVID-19 vaccines approved for use by at least one national regulatory authority [6] that are 50% to 95% effective in preventing the pandemic [7, 8]. Despite the occurrence of new variants being reported repeatedly, health authorities and vaccine manufacturers assure that the already-in-market vaccines still offer protection against most variants currently spreading [9]. This implies that vaccination programs have so far been the most successful strategy against COVID-19 pandemic.

Hesitancy towards the COVID-19 vaccine however severely impacted the prevalence of vaccination programs and consequently contributed to the burden COVID-19 has endangered. For example, in a study conducted by the imperial college of London, it is forecasted that high numbers of people refusing or delaying a vaccine could increase the mortality rate by up to eight times compared with ideal vaccination uptake [10]. In a similar study, it was also indicated that countries with broad populations refusing or delaying a COVID-19 vaccine could face death rates that are as much as nine times higher than in other populations.

With emerging research findings indicating that a substantial proportion of adults (especially in regions like Africa or the conservative section of the USA) are hesitant about a vaccine for COVID-19 [11, 12], important work is required to begin to understand and address this problem. It has been documented in large part over the years that vaccine hesitancy is a result of an inter-individual difference for example in personality, socio-economic status, demography and beliefs. The importance of identifying, describing, and understanding vaccine hesitant individuals as a key preparatory step for vaccine development is further emphasized by the World Health Organization’s (WHO, 2014) Strategic Advisory Group of Experts (SAGE) on Immunization [13]. It is imperative, therefore, that an effort is made to understand the multiple characteristics that define and distinguish those who are hesitant to a vaccine for COVID-19 from those who are accepting.

**Fig. 1:**
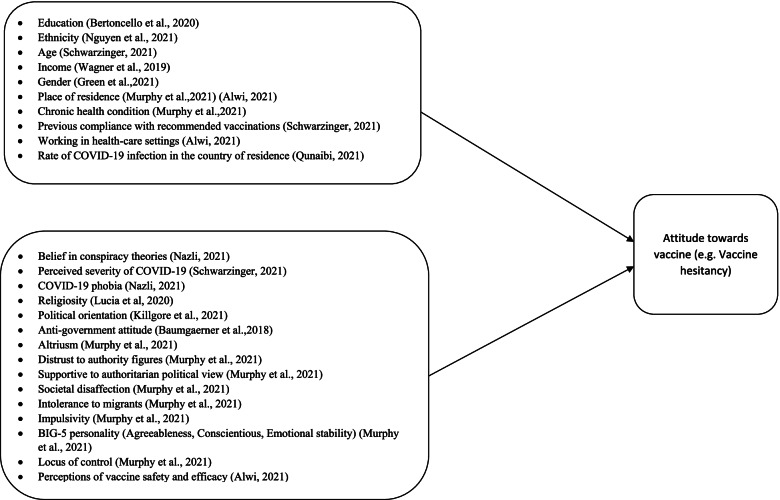
A review of Socio-demographic and Psychological factors that predict public’s attitude towards COVID-19 vaccine

In most of the cases, COVID-19 vaccine hesitancy is a result of belief in conspiracy theories. For example, from a poll of 1,640 people in the US, 28% of Americans believe that Bill Gates wants to use vaccines to implant microchips in people—with the figure rising to 44% among Republicans [14]. In another study conducted among 2032 Sub-Saharan African participants, about 7.3% of them believed that 5G technology was behind the COVID-19 pandemic [15]. Besides conspiracy theories, other health and socioeconomic variables also play significant role in predicting COVID-19 vaccine hesitancy. For example, COVID-19 vaccine hesitancy is most expressed by people who are less educated [16], racial and ethnic minority [17], less incomed [18], women [19], pregnant, city dwellers, and those suffering from an underlying chronic health problem [20]. A number of psychological constructs have also been explored in relation to COVID-19 vaccine hesitancy. For example, individuals who are -religious [21], politically conservative [22]and have an anti-government view [23] were shown to be vaccine hesitant. Moreover, individuals who are more—self-interested, distrustful of experts and authority figures (i.e. scientists, health care professionals, the state), in favor of authoritarian political views has more vaccine hesitant attitudes. Furthermore, societal disaffection, intolerance of migrants, impulsivity in ones thinking style, characteristics of disagreeability, emotional unstability, less conscientiousness, conviction that one’s lives are primarily under own control [20]—were shown to influence vaccine hesitancy (see Fig. 1).

Though an extensive and thorough investigation of different driving factors to COVID-19 vaccine hesitancy were reported, but the literature still misses some important factors that might predict COVID-19 vaccine hesitancy behaviors. First is individuals’ extreme reliance on traditional remedies. Especially in the African and Asian region, traditional remedies are considered to be a major source of treating illnesses [24]. In some cases as a first line of treatment [25]. As a result of this, individuals tend to underestimate or reject the complete use of modern medicine. Even in extreme cases, individuals tend to develop a disbelief to contrary modern medicine if they think there is a traditional remedy to replace it. Though we yet do not sufficiently know whether this also be the case to COVID-19 vaccine, but it was previously documented that people who use traditional, complementary and alternative medicine were found to be more vaccine hesitant [26].

Second, it was previously shown that the more people are religious, the more they tend to be COVID-19 vaccine hesitant [21]. But how useful are such pieces of information for a country where most of its population is considered to be highly religious (e.g. 95% religious in Indonesia) or very unreligious (e.g. 12% religious in France) [27]. For example, can we extrapolate from this finding to predict that the majority of the Indonesian (vs French) population is vaccine hesitant (vs non-hesitant) since the population is majorly religious (vs unreligious)? No, the recent vaccine-hesitancy prevalence findings does not confirm this prediction [28, 29]. This raises a need to study hesitancy against a more deeper classification of individuals than religiosity. For example, it is seemingly assumed that classifying individuals interms of their religious affiliations (denominations) instead of their religiosity is more precise in showing a more meaningful inter-individual difference than religiosity does [30]. For example, both a Methodist and an Evangelical score the same on religiosity, but as a result of their specific affiliations each end up showing different behaviors.

In this work, we investigated how individuals’ reliance on traditional remedies for treating COVID-19 compromises individuals’ willingness of taking COVID-19 vaccine. Moreover, we investigated how affiliation to different Christianity denominations (e.g. being an Orthodox Christian versus Protestant Christian) differently associate with vaccine hesitancy. While doing this, we also made an effort to partial out the effect of other key variables that are previously investigated to relate with COVID-19 vaccine hesitancy.

## Materials and methods

### Study setting

The study was conducted at Jimma University (JU), the largest and comprehensive public research university in Ethiopia located in the Jimma zone-Southwestern region of Ethiopia. The university operates four campuses and educates more than 43,000 students in 56 undergraduate and 103 postgraduate programs in regular, summer and distance education programs.

### Study design, participants and sampling

The source population for this study are all individuals who are staffs or students at Jimma University. Individuals aged ≥ 18 years and who are staffs or students at Jimma University were included in this study. Individuals who are already vaccinated for COVID-19 vaccine were excluded.^1^

Stratified simple random sampling technique was used to select study participants. First, stratification was done based on the faculty of staffs and students. Nine study faculties were identified. Then, four faculties were selected using lottery method and the sample was proportionally allocated. Finally, study participants were selected randomly and requested to fill the survey questionnaire.

### Sample size calculation

Single population proportion formula was used to calculate the sample size with the following assumptions: *P* (62.3%, the average proportion of intention to take vaccine among three Ethiopian towns [31]),d (the permissible Margin of error 5%) and Zα/2 corresponding to 95% confidence level.

$$n=\frac{{{(Z}_{a/2})}^{2}*p(1-p)}{{d}^{2}}=\frac{{(1.96)}^{2}0.37*0.62}{{0.05}^{2}}=340$$

Assuming a potential non-response rate of 5%, we increased the calculated sample size by 5%. The total number of individuals approached to attain desired sample were therefore 358 (340 + 5%*340). 

### Data collection tool, quality control, and procedure

The data was collected using both web-based and paper-based questionnaire.^2^ The web-based questionnaire was prepared on an online platform named Qualtrics and the survey link was distributed to targets through mail. The questionnaire involved structured and self-administered questions. The questionnaire was prepared both in English and Amharic formats and participants filled the questionnaire with the language of their convenience. The questionnaire tool consisted questions that measured socio-demographic characteristics, media exposure, attitude and knowledge about COVID-19 and COVID-19 vaccine, and COVID-19 vaccine hesitancy. Automatic and default attention check questions were included on the web-based version of the questionnaire to monitor and control participants who tried to fill the survey questionnaire randomly. In addition, participant’s duration of survey filling time was recorded so as to reject replies that spend less time than the mean duration of the majority of participants. The data collection instrument was pre-tested on Jimma University Medical Faculty students (on 2% of the sample size).

The pretest helped identify questions that create misunderstanding and questions where participants are uncomfortable to answer. Questions for political affiliation and race received small number of responses in the pretest implying participant discomfort on responding to these questions. The main study therefore avoided to include these questions. Moreover, we used the pretest responses to test the consistency of the translation. Participants in the pretest pilot study filled both the English and Amharic Questionnaire. The analysis of their responses on the two scales was highly correlated (*r* > 0.9) implying the consistency of the questionnaire translation.

Questions under ‘Attitude and knowledge to COVID-19 vaccine’ measured individual’s level of understanding, extent of information, level of concern and overall attitude on topics related to COVID-19 vaccine. For example, it covered topics like COVID-19 vaccine efficacy, safety, side-effect and complications. It also measured preference to traditional remedies than COVID-19 vaccine. Measures were adopted and modified from previous literatures [32, 33]. The questions measured the extent of how strongly or weakly participants approve or disapprove 12 item statements made about the topics: for example, ‘*I believe I can prevent or treat COVID-19 with traditional remedies than the C*OVID*-19 vaccine*’, ‘*I do not have enough information regarding COVID-19 vaccine*’, ‘ *I have concern with COVID-19 vaccine side effects*’, ‘*I believe that the COVID-19 vaccine is not safe*’, ‘*I have concerns on COVID-19 vaccine due to religious reasons’*. Participants were asked to approve the statements on a five points likert scale with 1 being ‘strongly disagree’ and 5 being ‘strongly agree’.

We operationally defined COVID-19 vaccine hesitancy as a delay in acceptance or refusal of vaccines despite availability of vaccine services. Measure is adopted from a previous literature [34]. Hesitancy was measured by asking participants how willing they would be to get a COVID-19 vaccine if it is freely offered to them. Response options include “definitely not willing,” “probably not willing,” “not sure,” “probably willing,” and “definitely willing.” For the primary outcome, responses into “willing” (definitely or probably willing) or “not willing” (all other responses) were dichotomized.

Participants were asked to indicate which religion they belong to out of four affiliations; ‘Orthodox Christian’, ‘Catholic Christian’, ‘Protestant Christian’, and ‘Muslim’ with the possibility of selecting ‘Others’ if they do not belong to one of these.

The study was conducted after receiving ethical approval from Jimma University Ethical Review Board (IRBJU/20/2021). Permission letter was obtained from Jimma University Institute of Health Ethical Review Board before data collection was started. Written informed consent was obtained from all participants after explaining the study’s purpose, risks, and benefits. Moreover, participants were assured the participation is entirely voluntary and personal information is not disclosed to third parties. The right to withdraw from the study was respected for participants.

### Statistical analyses

Data was exported from Qualtrics to the Statistical Package for Social Science (SPSS) version 28.0 for analysis. Frequency, percentage and mean was computed for descriptive statistics. The association between the independent and dependent variables was analyzed using the binomial logistic regression model. Bivariate analysis was done to select candidates for multivariate at *p* < 0.05. The adjusted model was run including all the sociodemographic, health condition and knowledge/attitude variables as independent variable. The fitness of the model was checked with Hosmer and Lemeshow (HL) test values. When running all the variables together as predictor, HL value is significant implying poor fit (χ2 (1,8) = 11.81, *p* = 0.044). However, removal of all nonsignificant variables from the adjusted analysis and rerunning the model yields a strong fit ( χ2 (1,8) = 2.5, *p* = 0.96)).

## Results

### Sociodemographic Characteristics of Respondents

A total of 338 participants were included in the current study, with a 94.4% response rate. More than 75% of respondents are in the age group of 18–29. Almost two-third (62.7%) of the respondents were males, three-fourth (67.8%) single, and more than half (63.9%) with a first degree. The overall vaccine hesitancy rate is 50%. Particularly, respondents with—age between 18 and 29, male and single, have a degree, Orthodox Christian, have more than 4 family members, and have a middle income—were found to be more hesitant to COVID-19 vaccine (See Table 1).

**Table 1:** Sociodemographic characteristics of participants (*n* = 338)

		Vaccine hesitancy		Total [338]
		No	Yes	
		N [169]	N [169]	
**Age**	18-29	125 (74)	133 (78.7)	258 (76.3)
	30-39	31 (18.3)	22 (13.0)	53 (15.7)
	40-49	9(5.3)	7 (4.1	16 (4.7)
	50-59	1(0.6)	5 (3.0)	6 (1.8)
	60 and above	3 (1.8)	2(1.2)	5 (1.5)
**Gender**	Male	105 (62.1)	107 (63.3)	212 (62.7)
	Female	64 (37.9)	62 (36.7)	126 (37.3)
**Marital Status**	Single	114 (67.5)	115 (68.0)	229 (67.8)
	Married	52 (30.8)	47 (27.8)	99 (29.3)
	Divorced	2 (1.2)	6 (3.6)	8 (2.4)
	Widowed	1 (0.6)	1(0.6)	2 (0.6)
**Educational level **	No formal education	2 (1.2)	3 (1.8)	5 (1.5)
	Elementary school	0 (0.0)	6(3.6)	6 (1.8)
	High school	7 (4.1)	10(5.9)	17 (5.0)
	Diploma	31 (18.3)	37(21.9)	68 (20.1)
	Degree	112 (66.3)	104 (61.5)	216 (63.9)
	Masters	11 (6.5)	3 (1.8)	14 (4.1)
	Doctorate degree	6 (3.6)	6 (3.6)	12 (3.6)
**Religion **	Orthodox Christian	63 (37.3)	69 (40.8)	132 (39.1)
	Catholic Christian	3(1.8)	4 (2.4)	7 (2.1)
	Protestant Christian	68(40.2)	48 (28.4)	116 (34.3)
	Muslim	28(16.6)	33 (19.5)	61 (18.0)
	Others	7 (4.1)	15 (8.9)	22 (6.5)
**Family members**	0 (Living alone)	23 (13.6)	25 (14.8)	48 (14.2)
	1	5 (3.0)	17 (10.1)	22 (6.5)
	2	18 (10.7)	18 (10.7)	36 (10.7)
	3	20 (11.8)	17 (10.1)	37 (10.9)
	4	35 (20.7)	29 (17.2)	64 (18.9)
	More than 4	68 (40.2)	63 (37.3)	131 (38.8)
**Monthly income **	Less than 1000 ETB	45 (26.6)	47 (27.8)	92 (27.2)
	From 1000 to 5500 ETB	62 (36.7)	77 (45.6)	139 (41.1)
	From 5500 to 6900 ETB	38 (22.5)	20 (11.8)	58 (17.2)
	6900 and above	24 (14.2)	25 (14.8)	49 (14.5)

### Associated Factors for vaccine hesitancy

On bivariate analysis, vaccine hesitancy has statistically significant associations with respondents’ -attitude and knowledge towards COVID-19 and COVID-19 vaccine, media exposure, monthly income and whether their family member has recently died with COVID-19 (See Tables 2, 3, 4). Regarding association with socioeconomic factors, the odds of becoming vaccine hesitant among middle income (monthly salary between 1000 to 5500 ETB^3^) is two times (AOR 2.17, 95% CI 1.05–4.5) more than those with a lower income (monthly salary of less than 1000 ETB; See Table 2). Furthermore, respondent’s extent of media exposure was associated with vaccine hesitancy (See Table 3). The odds of becoming vaccine hesitant among those whose source of COVID-19 information is from four media sources (Social Media, Mass Media, Health care worker and Friends/family/Neighbor) is 74% lower (AOR 0.26, 95% CI 0.09–0.69) than those with one media source. In addition, for respondents who reported death of a family member with COVID-19, the odds of becoming vaccine hesitant is seven times (AOR 6.9, 95% CI 1.8–26.4) more than those who does not have similar experience.

**Table 2:** Sociodemographic factors associated with vaccine hesitancy of study participants (*n* = 338)

		COR^5^ (95% CI)	***P*-value**	AOR^6^ (95% CI)	***P*-value**
**Age**	18–29				.57
	30–39	.67 (.36–1.2)	.185	.89 (.36 -2.2)	.82
	40–49	.73 (.26–2.0)	.546	.87 (.19 -4.04)	.86
	50–59	4.7 (.54 -40.7)	.160	5.9 (.29 -122)	.25
	60 and above	.63 (.1 -3.8)	.612	.08 (.00–10.1)	.32
**Gender**	Male				
	Female	.95 (.61 -1.47)	.82	.74 (.39 -1.4)	.36
**Marital Status**	Single				.38
	Married	.89 (.56 -1.43)	.65	.69 (.31 -1.5)	.38
	Divorced	2.97 (.59–15.0)	.19	3.8 (.38 -37.2)	.25
	Widowed	.99 (.06–16.0)	.99	.09 (.0 -70.6)	.48
**Educational level**	No formal education				.68
	Elementary school		.99		.99
	High school	.95 (.12–7.2)	.96	2.47 (.01 – 603)	.75
	Diploma	.79 (.12–5.1)	.81	1.00 (.00 – 4)	1.0
	Degree	.62 (.10–3.7)	.60	.95 (.00 -26)	.98
	Masters	.18 (.02 -1.6)	.13	.34 (.00–11)	.71
	Doctorate degree	.67 (.08 -5.5)	.71	1.57 (.00 – 4)	.87
**Religion**	Orthodox Christian				
	Catholic Christian	1.22 (1.2-.26)	.80	.46 (.04 -4.9)	.52
	Protestant Christian	.65 (.65-.39)	.09	.86 (.43 -1.7)	.68
	Muslim	1.07 (1.0-.58)	.81	1.96 (.88 -4.3)	.10
	Others	1.95 (1.9 -.75)	.17	2.16 (.56 -8.2)	.26
**Family members**	0 (Living alone)				
	1	3.1 (.9 -9.8)	.05	3.54 (.78 -16)	.10
	2	.92 (.38 -2.1)	.85	.51 (.15 -1.76)	.29
	3	.78 (.33 – 1.8)	.58	.51 ( .14 -1.7)	.29
	4	.76 (.36–1.6)	.48	.73 (.25 -2.1)	.57
	More than 4	.85 (.4 -1.6)	.64	.66 (.25 -1.7)	.39
**Monthly income**	Less than 1000 ETB				
	From 1000 to 5500 ETB	1.18 (.7 – 2.0)	.52	2.17 (1.05–4.5)	.04*
	From 5500 to 6900 ETB	.50 (.25—.99)	.05	.52 (.19 -1.4)	.19
	6900 and above	.99 (.49 -1.9)	.99	1.94 (.67 -5.5)	.22
**Media exposure**	Exposed to 1 source				
	Exposed to 2 sources	.92 (.53–1.59)	.76	.63 (.29–1.3)	.23
	Exposed to 3 sources	1.16 (.56 -2.43)	.68	1.1 (.4 -2.9)	.84
	Exposed to 4 sources	.51 (.26 – 1.02)	.06	.26 (.09 -.69)	.01**

*(*p* < 0.05), **(*p* < 0.01)

**Table 3:** Health condition factors associated with vaccine hesitancy of study participants (*n* = 338)

		**COR (95% CI)**	***P*-value**	**AOR (95% CI)**	***P*-value**
**Chronic disease**	No				
	Yes	1.70 (.82—3.52)	.15	2.3 (.77 -6.7)	.14
**Perceived healthiness**	Poor				
	Fair	1.78 (.1 -30.1)	.69	4.5 (.00 -93)	.69
	Good	.69 (.04 – 11.28)	.79	.85 (.00 -17)	.96
	Excellent	1.07 (.06- 17.37)	.96	2.8 (.00 -5)	.79
**Diagnosed with Covid-19**	No				
	Yes	.82 (.34–1.96)	.66	.45 (.1 -1.9)	.29
**Tested for Covid-19**	No				
	Yes	.85 (.54–1.34)	.48	1.3 (.65- 2.49)	.47
**Family diagnosed with Covid-19**	No				
	Yes	.87 (.53–1.45)	.61	.58 (.26 -1.3)	.18
**Family died with Covid-19**	No				
	Yes	1.96 (.81- 4.75)	.14	6.9 (1.8 -26.4)	.01**

**(*p* < 0.01)

Respondent’s attitude and knowledge about COVID-19 and COVID-19 vaccine was also associated with vaccine hesitancy (See Table 4). For example, respondents who have concern with COVID-19 vaccine side effects and respondents who believe that the COVID-19 vaccine is not effective have a 31% and 42% increase in the odds of becoming vaccine hesitant respectively than respondents who have less of those concerns. In the contrary, respondents who claimed that they do not have enough information regarding COVID-19 vaccine and respondents who believe that all COVID-19 vaccines in general are useful in controlling the COVID-19 pandemic have a 24% and 33% decrease in the odds of becoming COVID-19 vaccine hesitant respectively.

**Table 4:** Knowledge and attitude factors associated with vaccine hesitancy of study participants (*n* = 338)

	**COR (95% CI)**	***P*-value**	**AOR**^**7**^** (95% CI)**	***P*-value**
I do not have enough information regarding COVID-19 vaccine	.93 (.80–1.06)	.27	.76 (.62-.93)	.00**
I have concern with COVID-19 vaccine side effects	1.29 (1.07–1.53)	.00**	1.31(1.0–1.7)	.05*
I believe that the COVID-19 vaccine is not safe	1.42 (1.21 –1.67)	.00**	1.23 (.95–1.5)	.11
I think that the COVID-19 vaccine is not effective	1.64 (1.37–1.97)	.00**	1.42 (1.1 -1.8)	.01**
I think that COVID-19 is not any more dangerous	1.09 (.94–1.26)	.26	1.13 (.9 -1.4)	.29
I have fear of COVID-19 infection	.81 (.70-.94)	.00**	.82 (.66 -1.0)	.09
I am against vaccination in general	1.23 (1.03–1.47)	.02*	1.1 (.84–1.5)	.45
I have concerns on COVID-19 vaccine due to religious reasons	1.16 (1.0–1.34)	.04*	.99 (.7 -1.2)	.93
I have concerns on COVID-19 vaccine due to cultural reasons	1.06 (.91–1.23)	.48	.86 (.8 -1.1)	.26
I believe I can prevent or treat COVID-19 with traditional remedies than the Covid-19 vaccine	1.32 (1.13–1.54)	.00**	1.37 (1.0–1.7)	.01**
In general, I am concerned about serious complications of the COVID-19 vaccine	1.07 (.91–1.25)	.38	1.0 (.79–1.2)	.99
The COVID-19 vaccines, in general, will be useful in controlling the COVID-19 pandemic	.66 (.56—.79)	.00**	.67 (.52-.85)	.00**

*(*p* < 0.05), **(*p* < 0.01)

### Traditional remedies and COVID-19 vaccine hesitancy

30% (*N* = 105) of respondents agree that they can effectively prevent or treat COVID-19 with traditional remedies better than the COVID-19 vaccine. Consequently, such belief leads to a 37% increase in the odds of becoming vaccine hesitant (AOR 1.37, 95% CI 1.0–1.7).

### Religion and COVID-19 vaccine hesitancy

**Fig. 2:**
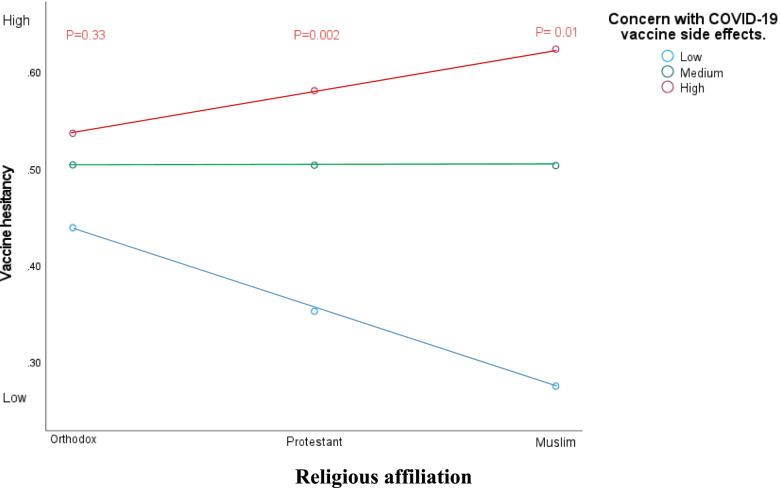
Religious affiliation moderating the relationship between Concern with COVID-19 side effects and Vaccine hesitancy

Participant’s distribution is diverse interms of religious composition. Orthodox Christian (39%; *n* = 132) and Protestant Christian (34%; *n* = 116) represent 75% of participants while those with Muslim affiliation account for 18% (*n* = 61). Vaccine hesitancy does not vary among the religious groups. However, the increase in the odds of becoming vaccine hesitant is not dependent on concern with COVID-19 side effects equally for all religious groups. For example, being concerned with COVID-19 side effects is not associated with vaccine hesitancy for Orthodox Christian participants but for Protestant Christian and Muslim participants (See Fig. 2).

## Discussion

Vaccine hesitancy is currently under-studied in developing countries [35]. It was found that half of the study population in this study are hesitant to vaccine. This prevalence is approximately similar to findings from other regional cities in Ethiopia like Sodo town (> 50%) and Gondor (44%) but way bigger than reported in the capital city Addis Ababa (19%). The magnitude of vaccine hesitancy increased as moving away from the capital city. A hypothesis for this variation in vaccine hesitancy across regions might have arised from inadequate information access about COVID-19 vaccine in smaller than bigger cities. Giving support to this hypothesis, a negative association was found between the frequency of media exposure and vaccine hesitancy. It was found that the more information access an individual has about COVID-19 vaccine from a variety of media sources (Social Media, Mass Media, Health care worker and Friends/family/Neighbor) than a single media source (one of the four), the odds of becoming vaccine hesitant decreased by 74%. Social media was previously represented as the flagship bearer of false information. Many previous studies [36] shown the susceptibility of social media in propagating unverified and scientifically unproven information about legit health services including COVID-19 vaccine, contributing to the prevalence of hesitancy. In our study, we document that vaccine hesitancy does not depend much on the type of the media but on the variety. This might imply that all media sources can be vulnerable to false information and to creating hesitancy. Therefore, a recommendable form of intervention in this regard is to use mix of media sources.

It is however important to be cautious of the accuracy of information pieces in media sources. This is because, use of several media sources precedes information overload, which negatively influences how individuals process information. A recent study [37] shown that information overload with COVID-19 made participant’s incline to greater heuristic and less systematic processing. Given individuals are more susceptible to process information automatically (vs critically) when exposed to a variety of media sources, this implies that exposure to COVID-19 information from a variety of media sources might not help in alleviating COVID-19 vaccine hesitancy if the sources hold some amount of disinformation.

Socioeconomic factors like monthly income have been shown to associate with COVID-19 vaccine hesitancy in previous literatures. However, a definite direction of relationship has not emerged. For example, while Soares [38] reported no association but several others [39] reported a negative association where individuals of lower income reported to be higher in COVID-19 vaccine hesitancy. Contrary to these previous findings, it is the middle incomed who are twofold more hesitant than the lower incomed in this study. It is our speculation that, since income was measured with an objective not subjective scale, middle incomed participants of this study might be regrouped as lower incomed within the income scale framework of previous studies in developed countries that has reported a negative association between income and hesitancy.

Immediately after the launch and implementation of the COVID-19 vaccination program, two problems arose that affected both the scientific community and the global public. First are mild to serious COVID-19 vaccine related side-effects which were reported and widely shared that even led to the ban of some vaccine brands from the market. For example, frequent reports of thromboembolism [40] has led to the ban of the Oxford–AstraZeneca COVID-19 vaccine in many European countries. Second confusion that came along with COVID-19 vaccine is the vulnerability of being reinfected even after being fully vaccinated. The prevalence of this event is 3 out of 100: out of 100 fully vaccinated, three get infected [41]. The consequences of these two confusions, side-effects and occurrence of infection, is long-range. It does not only prolong the burden of the pandemic or merely disrupt the global vaccine supply chain, but also contribute to more people becoming concerned about the COVID-19 vaccine. Our study brought an empirical evidence to this discourse by documenting an association between vaccine hesitancy and individuals concern on COVID-19 vaccine effectiveness and concern on COVID-19 vaccine side effects (Table 2). More interestingly, this association does not hold for those with Orthodox Christian affiliation: the magnitude of how concerned this group of participants are with COVID-19 vaccine side effects does not vary their hesitancy on the vaccine (Fig. 2). This might indicate the presence of other more important predictors of vaccine hesitancy in this group of population.

Amidst uncertainty and frustrations both in the pathophysiology and the management of COVID-19, many, especially in Africa, resorted to home remedies as immediate alternative or first line of action [42] to COVID-19. With home remedies treating mild COVID-19 like symptoms, it is expected that this further strengthens individuals reliance to traditional remedies more than on COVID-19 vaccine. As per the extent of our literature search, previous studies overlooked how such reliance on alternative treatments for COVID-19 contributes to the overall tendency to vaccine hesitancy.

The findings from this study will have practical contributions that might help in policy and health intervention activities. First, it is alarming that a significant proportion of the study population is hesitant to COVID-19 vaccine. Given that this figure is documented from a sample of an academic institution that involved more than 67% participants with atleast a first degree, it is daunting to predict that the figure might even plummet among other members of the society that are less educated. Global figures of COVID-19 vaccine hesitancy among universities is considerably lower than found in this study. For example, the prevalence is 24% [43] in an Italian university, 7.4% in a Czech university [44], 13% in a Qatari education sector population [45]. No similar data is reported from African universities than this particular study. If the most educated group of the society are hesitant to vaccines to this level, then Ethiopian health authorities might need to devise a strategic communication. A communication that leverages on the factors that are presumed to have caused the hesitancy. This could involve usage of every available source of media to disseminate legit and accurate COVID-19 and COVID-19 vaccine related information. Moreover, the communications should involve clinical presentations that document the minimal prevalence of vaccine side effects globally. Furthermore, communication strategies should be able to clearly indicate the value of being vaccinated. For example, future campaigns aimed at promoting COVID-19 vaccination intention could craft messages that depict the significantly reduced extent of disease severity in those vaccinated than non-vaccinated, despite there is still a chance of reinfection after full vaccination. Moreover, religious affiliation can also be used as an important characteristics to segment target groups when running anti-vaccine-hesitancy communications.

### Limitations

Our study should be interpreted within the context of its limitations. First, our study population was restricted to an educational institution that constitutes individuals with atleast high school or above high school educational qualifications. This limits the generalizability of our study findings to the general population. Second, our study being cross-sectional in design limits inference of causal relationships between the identified factors and vaccine hesitancy.

## Conclusion

Though vaccination has proven to be one of the best means to bring an end to the pandemic, but behaviors like hesitancy are a challenge to its implementation. A limited exposure to media information, concerns about COVID-19 vaccine effectiveness and side-effect and believing that one can better treat COVID-19 with a traditional remedy – related significantly with the hesitancy tendency. Health authorities therefore require to devise a communication effort that leverages on these factors.

## Supplementary Information

**Additional file 1.** 

**Additional file 2.** 

## Data Availability

The data is available from https://osf.io/5yazm/?view_only=e2c4cc276ec24d0688958a8520d07cba. The data is publicly available for use.

## Footnotes

1 Inclusion and Exclusion decision was made based on participants responses for questions on the first section of the Survey (Age, Vaccination Status and Consent). Participants aged > 18 and those who mentioned they are not vaccinated were allowed to proceed with the main survey.

2 We first tried to reach staffs and students at the 4 faculties through a non-targeted institutional based mail. Since the turnout of the online collection wasn’t effective, we switched to paper-based collection with a data collector.

3 ETB = Ethiopian Birr. 1,000 ETB is equivalent to 19.46 USD as of 01.05.2022.
