# Supplementary material for: Understanding determinants of COVID-19 vaccine hesitancy; an emphasis on the role of religious affiliation and individual’s reliance on traditional remedy
Source: BMC Public Health. 2022 Jun 7;22:1142. doi: 10.1186/s12889-022-13485-2 (PMC9172606; doi:10.1186/s12889-022-13485-2)
Supplement: Supplementary file 1 — Additional file 1. [file 12889_2022_13485_MOESM1_ESM.pdf]

Attitude to the Covid-19 pandemic and the Covid-19 vaccine

Consent

INSTRUCTIONS:
We are asking you to participate in a research study titled “attitude towards COVID-19 vaccine”. We will describe this study to you and answer any of your questions.

What the study is about
The purpose of this research is to better understand people’s attitude and knowledge about the COVID-19 pandemic and the COVID-19 vaccine.

What we will ask you to do
We will ask you to answer close ended questions. The study should take approximately 3-5 minutes to complete.

Risks and discomforts
We do not anticipate any risks from participating in this research.

Benefits
Information from this study may help us learn more about people’s attitude towards the COVID-19 pandemic and the COVID-19 vaccine.

Privacy/Confidentiality/Data Security
This is an anonymous survey, there is no identifying information we collect from you.

Sharing De-identified Data Collected in this Research
De-identified data from this study may be shared with the research community at large to advance science and health. We will remove or code any personal information that could identify you before files are shared with other researchers to ensure that, by current scientific standards and known methods, no one will be able to identify you from the information we share.

Taking part is voluntary
Your involvement is voluntary, you may refuse to participate before the study begins, and you may discontinue at any time. This survey was designed as a package so that it would be most helpful if you would answer every question unless any of them make you uncomfortable or cause any stress. Note that you have the right not to answer any questions that are stressful or cause discomfort.

If you have questions
The main researcher conducting this study is Hanna Defar Hassen, a medical intern at Jimma University College of Health Sciences. If you have questions later, you may contact Hanna

Defar Hassen at hannadefh@gmail.com or at 0912780966. If you have any questions or concerns regarding your rights as a participant in this study, you may contact Jimma University Ethics Review Committee at 0911409074 or https://healthresearchweb.org/en/ethiopia/ethics_1066.

Statement of Consent

- ○ Yes, Continue to Survey
- ○ No, I don't want to take part in this Survey

Age (Please mark one answer)

- ○ 18-29
- ○ 30-39
- ○ 40-49
- ○ 50-59
- ○ 60 and above

Gender (Please mark one answer)

- ○ Male
- ○ Female

Marital status (Please mark one answer)

- ○ Single
- ○ Married
- ○ Divorced
- ○ Widowed

Educational level (Please mark one answer)

- ○ No formal education
- ○ Elementary school
- ○ High school
- ○ Diploma
- ○ Degree
- ○ Masters
- ○ Doctorate degree

Religion (Please mark one answer)

- ○ Orthodox Christian
- ○ Catholic Christian
- ○ Protestant Christian
- ○ Muslim
- ○ Others

With how many family members are you living with (Please mark one answer)?

- ○ 0 (living alone)
- ○ 1
- ○ 2
- ○ 3
- ○ 4
- ○ More than 4

Monthly income (Please mark one answer)

- ○ Less than 1000 ETB
- ○ 1000-5500 ETB
- ○ 5500-6900 ETB
- ○ Above 6900 ETB

Do you have any chronic disease (a disease is chronic when it requires life-long treatment, for example E.g., Diabetics, hypertension, asthma, etc)?

- ○ Yes
- ○ No

How would you rate your health overall? Would you say that your health is excellent, good, fair, or poor?

- ○ Poor
- ○ Fair
- ○ Good
- ○ Excellent

Do you have any smoking habits (Please mark one answer)?

- ○ Yes
- ○ No

Please respond to the following questions?

	Yes	No
Have you been diagnosed with Covid-19 before?	○	○
Have you ever tested for Covid-19 previously ?	○	○
Is any of your family member or friend ever diagnosed with COVID-19?	○	○
Has any of your family member or friend died due to COVID-19?	○	○
Have you already vaccinated for Covid-19?	○	○

What is your source of information regarding COVID-19 (Please mark one or more answers)?

- ☐ Social media (WhatsApp, Telegram, Twitter, Instagram, Facebook, Website etc)
- ☐ Mass media (TV, Radio, Newspaper)
- ☐ Healthcare worker
- ☐ Friends, family, neighbor etc

How willing are you to get a COVID-19 vaccine if it is freely offered to you?

- ○ Definitely not willing
- ○ Probably not willing
- ○ Not sure
- ○ Probably willing
- ○ Definitely willing

The following 12 items measure your opinion, knowledge or attitude about Covid-19 vaccine. For each item, please choose the option based on how much you agree on each

statement (1=strongly disagree if you think you do not agree with the statement; 5=Strongly agree if you think you do agree with the statement).

	Strongly disagree	Somewhat disagree	Neither agree nor disagree	Somewhat agree	Strongly agree
I do not have enough information regarding COVID-19 vaccine.	○	○	○	○	○
I have concern with COVID-19 vaccine side effects.	○	○	○	○	○
I believe that the COVID-19 vaccine is not safe.	○	○	○	○	○
I think that the COVID-19 vaccine is not effective.	○	○	○	○	○
I think that COVID-19 is not any more dangerous.	○	○	○	○	○
I have fear of COVID-19 infection.	○	○	○	○	○
I am against vaccination in general.	○	○	○	○	○
I have concerns on COVID-19 vaccine due to religious reasons.	○	○	○	○	○
I have concerns on COVID-19 vaccine due to cultural reasons.	○	○	○	○	○

I believe I can prevent or treat COVID-19 with traditional remedies than the Covid-19 vaccine.	○	○	○	○	○
In general, I am concerned about serious complications of the COVID-19 vaccine.	○	○	○	○	○
The COVID-19 vaccines, in general, will be useful in controlling the COVID-19 pandemic.	○	○	○	○	○
