# Supplementary material for: Understanding determinants of COVID-19 vaccine hesitancy; an emphasis on the role of religious affiliation and individual’s reliance on traditional remedy
Source: BMC Public Health. 2022 Jun 7;22:1142. doi: 10.1186/s12889-022-13485-2 (PMC9172606; doi:10.1186/s12889-022-13485-2)
Supplement: Supplementary file 2 — Additional file 2. [file 12889_2022_13485_MOESM2_ESM.pdf]

Age	Gen	MaritalSta	Education	Rel	relD	rel3	FamilyMer	Income	ChrDis	PercHeal	SmokingBe	CovidDiagr	CovidTest
2	1	3	3	4	#NULL!	3,00	6	1	0	3	1	1	1
1	1	1	4	5	#NULL!	#NULL!	2	4	0	4	0	0	1
1	1	2	4	1	1,00	1,00	5	2	0	4	0	0	1
1	2	2	4	3	0,00	2,00	3	2	0	4	0	0	0
1	1	1	4	1	1,00	1,00	1	2	0	4	0	0	0
1	1	1	4	3	0,00	2,00	5	1	0	2	0	0	0
1	1	1	4	3	0,00	2,00	5	1	0	2	0	0	0
1	2	2	4	3	0,00	2,00	2	3	0	4	0	0	1
1	1	1	4	1	1,00	1,00	6	1	0	#NULL!	0	0	0
1	2	1	4	3	0,00	2,00	5	2	0	4	0	0	1
1	1	1	4	4	#NULL!	3,00	6	1	0	4	0	0	0
1	1	1	3	1	1,00	1,00	4	2	0	4	0	0	0
2	2	2	3	1	1,00	1,00	6	2	0	4	0	0	1
1	1	1	4	3	0,00	2,00	6	4	0	4	0	0	1
1	1	1	4	3	0,00	2,00	1	3	0	4	0	0	0
2	1	2	5	3	0,00	2,00	3	3	0	3	0	0	1
1	2	1	5	4	#NULL!	3,00	1	2	0	4	0	0	1
1	2	2	4	1	1,00	1,00	5	3	1	4	1	0	1
1	2	2	4	4	#NULL!	3,00	5	2	0	4	0	0	0
1	1	1	4	1	1,00	1,00	1	4	0	4	0	0	1
1	2	1	4	4	#NULL!	3,00	6	2	0	3	0	0	0
1	2	1	3	4	#NULL!	3,00	5	1	1	3	0	0	1
1	1	1	4	4	#NULL!	3,00	3	2	0	2	0	0	0
1	2	1	4	4	#NULL!	3,00	6	2	1	3	0	0	1
1	2	1	4	4	#NULL!	3,00	6	2	1	3	0	0	1
1	1	1	4	4	#NULL!	3,00	3	1	0	4	0	0	0
2	1	1	4	1	1,00	1,00	1	3	0	4	0	0	1
1	1	1	4	1	1,00	1,00	6	4	1	3	0	0	0
1	1	1	4	3	0,00	2,00	3	3	0	3	0	0	0
1	1	1	4	5	#NULL!	#NULL!	1	2	0	4	0	1	1
1	2	2	4	4	#NULL!	3,00	5	2	0	4	0	0	0
1	2	1	4	1	1,00	1,00	6	1	0	4	0	0	0

1	1	1	4	1	1,00	1,00	6	2	0	3	0	0	0
1	1	1	4	4	#NULL!	3,00	3	1	0	3	0	0	1
1	2	1	4	4	#NULL!	3,00	6	1	0	4	0	0	0
1	1	1	4	3	0,00	2,00	1	2	0	4	0	0	0
1	1	1	4	4	#NULL!	3,00	3	1	0	4	0	0	1
2	2	2	3	3	0,00	2,00	6	2	0	4	0	1	1
4	2	4	3	5	#NULL!	#NULL!	1	2	1	3	0	0	0
2	1	1	5	5	#NULL!	#NULL!	1	4	0	3	0	0	0
1	2	1	4	3	0,00	2,00	5	1	0	3	0	0	0
1	1	3	3	1	1,00	1,00	6	1	1	3	1	0	0
2	1	2	1	1	1,00	1,00	3	1	0	4	0	0	0
1	1	1	3	1	1,00	1,00	4	1	1	2	0	0	0
1	1	1	4	1	1,00	1,00	5	1	1	4	1	0	1
1	1	1	4	1	1,00	1,00	5	1	0	4	0	0	1
1	1	1	4	5	#NULL!	#NULL!	6	1	0	3	0	0	0
1	2	2	2	1	1,00	1,00	4	2	0	3	0	0	0
1	1	1	4	3	0,00	2,00	6	2	0	4	0	0	0
1	1	1	4	3	0,00	2,00	1	1	0	4	0	0	0
1	2	1	4	5	#NULL!	#NULL!	6	2	0	4	0	0	0
1	1	1	4	3	0,00	2,00	6	2	0	4	0	0	0
1	1	1	4	3	0,00	2,00	5	3	0	4	0	0	0
1	1	1	4	3	0,00	2,00	6	2	0	4	0	0	0
1	1	1	4	3	0,00	2,00	4	2	0	4	0	0	0
1	1	1	4	3	0,00	2,00	5	2	0	4	0	0	0
1	2	1	4	4	#NULL!	3,00	5	1	0	3	0	0	0
1	1	2	1	1	1,00	1,00	5	2	0	4	0	0	0
2	1	2	3	4	#NULL!	3,00	3	2	0	4	0	1	1
1	1	1	4	3	0,00	2,00	6	1	0	4	0	0	0
1	1	2	4	3	0,00	2,00	3	2	0	4	0	0	0
1	2	1	0	4	#NULL!	3,00	2	1	1	2	0	1	0
3	1	2	3	3	0,00	2,00	3	2	1	2	0	0	1
1	2	1	4	1	1,00	1,00	6	3	0	4	0	0	0
1	1	1	4	1	1,00	1,00	1	4	0	4	0	0	0

1	2	1	3	1	1,00	1,00	6	1	0	4	0	0	1
1	1	2	5	4	#NULL!	3,00	2	4	0	3	0	0	0
2	1	1	6	2	#NULL!	#NULL!	5	4	0	3	0	0	0
1	2	1	4	1	1,00	1,00	6	1	0	4	0	0	0
1	2	1	4	1	1,00	1,00	6	1	0	4	0	0	0
1	2	1	2	1	1,00	1,00	2	1	0	3	0	0	0
1	1	1	3	1	1,00	1,00	5	3	0	2	0	0	0
1	2	1	4	3	0,00	2,00	1	2	1	3	0	1	1
2	2	2	4	1	1,00	1,00	6	4	0	4	0	0	0
1	1	1	4	3	0,00	2,00	4	1	0	3	0	0	1
4	1	2	4	4	#NULL!	3,00	6	3	0	3	0	0	0
2	1	1	3	1	1,00	1,00	1	2	0	4	0	0	0
1	1	1	4	3	0,00	2,00	6	1	0	3	0	0	1
1	1	1	4	1	1,00	1,00	3	1	0	2	0	0	0
1	1	1	4	4	#NULL!	3,00	4	1	0	4	0	0	0
5	1	3	0	3	0,00	2,00	5	2	1	2	0	0	1
1	2	1	3	1	1,00	1,00	6	1	0	4	0	0	0
1	2	1	3	3	0,00	2,00	6	1	0	4	0	0	0
1	2	1	3	3	0,00	2,00	6	1	0	4	0	0	0
2	2	3	3	1	1,00	1,00	6	2	1	3	0	0	1
1	2	2	2	3	0,00	2,00	5	1	0	4	0	0	0
1	1	2	4	3	0,00	2,00	4	2	0	2	0	0	0
2	1	2	4	3	0,00	2,00	5	2	0	3	0	0	0
2	1	1	1	2	#NULL!	#NULL!	2	2	1	2	0	0	0
2	1	1	3	1	1,00	1,00	5	2	0	2	0	0	0
1	1	1	3	1	1,00	1,00	1	2	0	3	0	0	0
2	1	2	3	4	#NULL!	3,00	3	2	0	4	0	0	1
1	1	1	4	1	1,00	1,00	5	4	0	4	0	0	0
1	2	2	3	1	1,00	1,00	4	2	1	2	0	0	0
2	1	1	4	3	0,00	2,00	5	2	0	4	0	0	0
4	2	2	3	3	0,00	2,00	6	4	1	3	0	0	0
1	1	1	3	3	0,00	2,00	6	1	0	4	0	0	0
3	1	2	4	3	0,00	2,00	6	2	0	4	0	0	0

1	1	2	3	1	1,00	1,00	4	2	0	4	0	0	0
1	1	1	3	1	1,00	1,00	5	3	0	2	0	0	0
1	1	1	4	3	0,00	2,00	2	4	0	3	0	0	0
1	2	1	3	1	1,00	1,00	1	2	0	2	0	0	0
1	1	2	4	1	1,00	1,00	4	2	0	2	0	0	0
1	1	1	3	1	1,00	1,00	5	2	0	4	0	0	0
1	1	2	3	1	1,00	1,00	2	2	0	2	0	0	0
1	2	2	3	1	1,00	1,00	4	1	0	3	0	0	1
4	2	2	3	1	1,00	1,00	6	4	0	4	0	0	0
3	2	2	4	1	1,00	1,00	6	2	0	4	0	0	0
1	1	1	4	2	#NULL!	#NULL!	1	2	0	4	0	0	0
1	1	1	4	3	0,00	2,00	1	2	1	3	0	0	0
1	1	2	3	1	1,00	1,00	3	2	1	2	0	0	0
2	1	1	4	3	0,00	2,00	1	3	0	4	0	0	1
1	2	3	3	4	#NULL!	3,00	3	4	0	3	0	0	0
2	2	2	4	4	#NULL!	3,00	5	2	0	2	0	0	0
1	1	1	4	1	1,00	1,00	1	2	0	4	0	0	0
1	1	1	5	3	0,00	2,00	1	3	0	4	0	0	0
2	1	2	4	3	0,00	2,00	5	4	0	3	0	0	0
2	1	2	4	4	#NULL!	3,00	6	2	0	3	0	0	0
2	1	1	2	1	1,00	1,00	5	1	0	3	0	0	0
2	1	1	3	1	1,00	1,00	1	2	0	4	0	0	0
2	1	1	2	1	1,00	1,00	3	2	0	3	0	0	0
1	2	2	4	4	#NULL!	3,00	3	3	0	4	0	0	0
2	2	2	4	4	#NULL!	3,00	5	2	0	4	0	0	0
3	1	2	4	4	#NULL!	3,00	6	4	0	2	0	0	0
1	1	1	4	1	1,00	1,00	1	1	0	2	0	0	0
3	2	2	3	3	0,00	2,00	6	1	0	4	0	0	1
1	2	1	6	3	0,00	2,00	1	3	1	2	0	0	1
1	2	1	4	1	1,00	1,00	6	1	0	4	0	0	0
1	2	2	3	3	0,00	2,00	6	1	0	4	0	0	1
1	2	2	4	3	0,00	2,00	1	2	0	4	0	0	1
1	2	2	1	3	0,00	2,00	4	1	0	4	0	0	0

1	2	1	4	1	1,00	1,00	6	3	0	4	0	0	1
1	1	1	4	1	1,00	1,00	6	2	0	4	0	0	1
1	1	1	4	4	#NULL!	3,00	6	2	0	3	0	0	0
1	1	1	4	4	#NULL!	3,00	6	1	0	2	0	0	0
1	1	1	4	4	#NULL!	3,00	6	1	0	3	0	0	1
5	1	4	6	4	#NULL!	3,00	6	1	0	2	0	0	1
3	1	2	4	3	0,00	2,00	5	2	0	4	0	0	0
2	1	1	4	1	1,00	1,00	4	2	0	4	0	0	0
2	1	1	3	1	1,00	1,00	3	3	0	2	0	0	0
5	1	2	0	5	#NULL!	#NULL!	6	1	1	3	0	0	0
1	1	1	4	3	0,00	2,00	4	1	0	3	0	0	0
1	1	1	4	1	1,00	1,00	1	1	0	3	0	0	0
1	1	1	4	1	1,00	1,00	5	2	0	3	0	0	0
1	2	1	4	3	0,00	2,00	6	1	1	3	0	0	0
1	1	1	4	4	#NULL!	3,00	6	2	0	3	0	0	0
1	1	1	4	4	#NULL!	3,00	6	1	0	4	0	0	1
1	2	1	4	4	#NULL!	3,00	6	1	0	4	0	0	0
1	1	1	4	1	1,00	1,00	6	2	0	3	0	0	0
1	2	1	4	1	1,00	1,00	6	2	0	3	0	0	0
1	2	1	4	4	#NULL!	3,00	6	1	0	4	0	0	0
1	1	1	4	3	0,00	2,00	6	1	0	4	0	0	0
1	1	2	4	1	1,00	1,00	4	3	0	4	1	0	0
1	2	2	4	3	0,00	2,00	6	1	0	4	0	0	0
1	1	1	3	1	1,00	1,00	2	2	0	2	0	0	0
2	2	2	3	3	0,00	2,00	6	3	0	4	0	0	0
1	1	1	6	4	#NULL!	3,00	1	3	0	3	1	0	0
2	1	2	4	4	#NULL!	3,00	4	3	0	4	0	0	0
1	1	1	2	4	#NULL!	3,00	6	1	0	3	0	0	0
1	1	1	3	1	1,00	1,00	6	2	0	4	0	0	0
1	2	1	4	1	1,00	1,00	5	2	0	3	0	0	0
1	1	2	5	3	0,00	2,00	4	4	0	3	0	0	0
1	1	1	4	3	0,00	2,00	6	2	0	4	0	0	0
1	1	1	4	1	1,00	1,00	6	1	0	4	0	0	0

1	1	1	4	1	1,00	1,00	4	1	0	4	0	0	1
1	1	1	4	1	1,00	1,00	6	2	1	2	0	0	0
1	1	1	4	3	0,00	2,00	5	2	0	4	0	0	0
1	1	1	4	1	1,00	1,00	4	1	0	4	0	0	0
1	1	1	0	5	#NULL!	#NULL!	1	1	1	1	1	0	1
1	1	1	4	1	1,00	1,00	6	1	0	4	0	0	0
1	2	1	2	1	1,00	1,00	5	3	0	2	0	0	0
2	2	2	4	1	1,00	1,00	3	4	0	3	0	0	0
2	2	2	3	3	0,00	2,00	3	2	1	3	0	0	0
1	2	1	4	4	#NULL!	3,00	6	1	0	4	0	0	0
2	2	2	4	1	1,00	1,00	6	3	0	3	0	0	0
1	2	1	4	2	#NULL!	#NULL!	6	2	0	2	0	0	0
1	2	1	4	2	#NULL!	#NULL!	6	2	0	3	0	0	1
2	2	2	4	3	0,00	2,00	5	3	1	2	0	0	1
2	2	2	3	4	#NULL!	3,00	5	3	0	3	0	0	0
1	2	1	3	1	1,00	1,00	6	1	0	2	0	1	1
1	2	1	6	1	1,00	1,00	6	3	0	3	0	0	0
1	2	1	4	3	0,00	2,00	6	2	0	4	0	0	0
1	2	1	4	3	0,00	2,00	6	4	0	4	0	0	0
1	2	1	1	1	1,00	1,00	6	2	0	3	0	0	0
1	2	1	3	3	0,00	2,00	6	2	0	3	0	0	1
1	2	1	3	3	0,00	2,00	5	2	0	3	0	0	0
1	2	1	4	4	#NULL!	3,00	6	1	0	2	0	0	1
3	2	2	3	3	0,00	2,00	6	2	1	3	0	0	0
2	2	2	3	4	#NULL!	3,00	4	4	0	3	0	0	1
1	2	1	4	1	1,00	1,00	6	2	0	4	0	0	1
1	2	1	3	4	#NULL!	3,00	2	2	1	3	0	0	0
1	2	1	4	3	0,00	2,00	5	2	0	3	0	0	0
1	2	2	4	1	1,00	1,00	4	2	0	2	0	0	0
2	2	1	4	3	0,00	2,00	6	4	0	4	0	0	1
4	2	3	4	3	0,00	2,00	4	4	0	3	0	0	0
3	2	3	3	1	1,00	1,00	4	2	1	2	0	0	1
1	1	1	3	4	#NULL!	3,00	1	2	0	3	1	0	0

1	1	1	4	1	1,00	1,00	3	2	0	4	0	0	0
1	1	1	4	1	1,00	1,00	6	1	0	2	1	1	1
1	1	1	4	1	1,00	1,00	6	1	0	3	0	0	0
1	1	1	3	1	1,00	1,00	1	2	0	4	0	0	0
1	1	1	4	4	#NULL!	3,00	6	2	0	4	0	0	0
1	1	1	4	3	0,00	2,00	6	2	0	4	0	0	1
1	1	1	4	3	0,00	2,00	5	2	0	4	0	0	0
1	1	2	4	3	0,00	2,00	3	3	0	2	0	0	0
1	1	2	4	3	0,00	2,00	2	2	0	4	0	0	0
1	2	1	4	3	0,00	2,00	6	4	0	3	0	0	1
1	2	1	4	3	0,00	2,00	5	1	0	4	0	1	1
1	2	1	4	4	#NULL!	3,00	5	4	0	2	0	0	0
1	1	1	4	4	#NULL!	3,00	6	1	0	3	0	0	1
1	1	1	3	3	0,00	2,00	6	2	0	3	0	0	0
1	1	1	4	3	0,00	2,00	6	2	0	4	0	0	0
1	1	2	3	3	0,00	2,00	4	2	0	4	0	0	1
1	1	1	4	1	1,00	1,00	5	2	0	3	0	0	0
2	1	2	3	4	#NULL!	3,00	2	1	0	4	0	0	0
1	1	1	4	1	1,00	1,00	6	1	0	4	0	0	0
1	1	1	4	4	#NULL!	3,00	6	1	0	4	0	0	0
1	1	2	5	3	0,00	2,00	5	4	0	4	0	0	1
1	2	1	4	3	0,00	2,00	5	1	0	3	0	0	1
1	1	1	4	3	0,00	2,00	6	3	0	3	0	0	0
1	1	1	4	1	1,00	1,00	2	1	0	4	0	0	1
1	1	1	2	1	1,00	1,00	5	2	0	3	0	0	1
2	1	2	3	1	1,00	1,00	6	2	0	4	0	0	0
2	2	2	4	3	0,00	2,00	5	1	0	4	0	0	1
3	2	1	3	1	1,00	1,00	5	3	0	3	0	0	0
1	1	2	4	2	#NULL!	#NULL!	3	2	0	4	0	0	0
2	1	2	4	3	0,00	2,00	5	2	0	3	0	0	1
1	1	1	4	3	0,00	2,00	6	1	0	4	0	0	0
1	1	2	3	1	1,00	1,00	4	3	0	4	0	0	0
1	1	1	4	1	1,00	1,00	6	2	0	4	0	0	0

1	1	1	4	1	1,00	1,00	6	1	0	4	0	0	0
1	1	1	6	1	1,00	1,00	6	1	0	4	0	0	1
1	1	1	6	3	0,00	2,00	6	1	0	3	0	0	0
1	2	1	4	3	0,00	2,00	4	3	1	4	0	0	0
1	1	1	4	1	1,00	1,00	1	2	0	4	0	0	0
1	1	1	4	5	#NULL!	#NULL!	6	2	0	4	0	0	0
1	1	1	2	1	1,00	1,00	6	1	0	3	0	0	0
2	1	2	6	5	#NULL!	#NULL!	2	4	0	3	0	0	1
1	1	1	2	5	#NULL!	#NULL!	6	2	0	4	0	0	0
1	1	1	6	1	1,00	1,00	6	3	0	4	0	1	0
1	1	1	6	1	1,00	1,00	1	4	0	4	0	0	1
1	1	1	2	5	#NULL!	#NULL!	6	3	0	4	0	0	0
1	2	1	4	5	#NULL!	#NULL!	6	2	0	3	0	0	0
1	1	1	4	5	#NULL!	#NULL!	6	4	0	4	0	0	0
1	1	1	4	3	0,00	2,00	6	1	0	4	0	0	1
4	1	1	4	5	#NULL!	#NULL!	6	3	0	4	0	0	1
1	1	1	6	5	#NULL!	#NULL!	6	4	0	3	0	0	0
1	2	2	4	5	#NULL!	#NULL!	3	2	0	4	0	0	0
1	2	1	4	3	0,00	2,00	5	2	0	4	0	0	0
1	1	1	4	1	1,00	1,00	2	3	0	3	1	0	0
1	1	1	4	3	0,00	2,00	6	4	0	4	0	0	0
2	2	2	4	4	#NULL!	3,00	6	4	0	4	0	0	1
3	1	2	5	3	0,00	2,00	6	4	0	4	0	1	1
1	2	1	4	1	1,00	1,00	5	4	0	2	0	0	0
1	1	1	4	4	#NULL!	3,00	6	3	0	3	0	0	0
1	1	1	4	4	#NULL!	3,00	5	1	0	4	0	0	0
1	1	2	4	3	0,00	2,00	2	2	0	4	0	1	1
1	2	1	4	4	#NULL!	3,00	3	2	0	4	0	0	1
1	1	1	4	5	#NULL!	#NULL!	6	2	0	4	0	0	0
1	2	1	4	3	0,00	2,00	2	2	0	4	0	0	0
1	1	1	5	1	1,00	1,00	1	4	0	3	0	0	0
2	1	2	4	3	0,00	2,00	6	4	0	3	0	0	0
3	1	2	4	3	0,00	2,00	5	4	0	3	0	0	0

1	1	1	4	3	0,00	2,00	1	4	0	4	0	0	1
2	1	2	3	1	1,00	1,00	4	4	0	4	0	0	1
1	1	2	2	1	1,00	1,00	3	2	0	3	0	0	0
1	1	1	4	4	#NULL!	3,00	5	2	0	4	0	0	0
1	2	2	4	5	#NULL!	#NULL!	5	4	1	2	0	0	0
2	1	2	4	3	0,00	2,00	5	4	0	4	0	0	0
1	1	1	4	3	0,00	2,00	6	2	0	3	0	0	0
1	1	2	4	3	0,00	2,00	5	2	0	4	0	0	1
1	2	1	4	1	1,00	1,00	6	1	0	4	0	0	0
1	1	1	4	1	1,00	1,00	1	1	0	3	0	0	0
1	1	1	4	3	0,00	2,00	6	1	0	4	0	0	1
1	1	1	4	1	1,00	1,00	3	2	0	2	0	0	0
1	1	1	4	3	0,00	2,00	6	1	0	4	0	0	0
1	1	1	4	1	1,00	1,00	1	3	0	4	0	0	1
1	1	1	4	3	0,00	2,00	2	2	0	4	0	0	0
1	1	1	4	1	1,00	1,00	1	4	0	4	0	1	1
1	1	2	4	1	1,00	1,00	5	4	0	4	0	0	1
1	1	2	4	4	#NULL!	3,00	5	2	0	4	0	0	0
1	2	1	4	3	0,00	2,00	5	4	0	4	0	0	1
1	1	1	4	3	0,00	2,00	6	1	0	4	0	0	0
1	1	1	4	5	#NULL!	#NULL!	1	3	0	2	1	0	0
1	2	1	3	4	#NULL!	3,00	6	2	0	4	0	0	0
1	1	1	4	1	1,00	1,00	4	2	0	3	0	0	0
2	1	1	5	3	0,00	2,00	1	3	0	3	0	0	0
2	1	1	4	3	0,00	2,00	1	3	0	4	0	0	1
1	2	1	4	5	#NULL!	#NULL!	1	3	0	3	0	1	1
3	2	2	3	1	1,00	1,00	5	4	0	3	0	0	1
1	2	1	5	4	#NULL!	3,00	6	4	0	4	0	0	0
1	1	1	4	3	0,00	2,00	6	1	0	4	0	0	0
1	1	1	4	3	0,00	2,00	4	1	0	4	0	0	0
2	2	2	1	2	#NULL!	#NULL!	4	1	0	2	0	0	1
1	2	1	4	1	1,00	1,00	1	3	0	4	0	0	0
1	2	1	4	1	1,00	1,00	4	3	0	4	0	1	1

3	2	2	4	1	1,00	1,00	6	4	0	3	0	0	1
1	1	1	4	1	1,00	1,00	6	2	0	3	0	0	0
3	1	2	3	4	#NULL!	3,00	1	1	0	2	0	1	1
1	1	1	4	3	0,00	2,00	3	3	0	4	0	0	0
1	1	1	4	1	1,00	1,00	3	3	0	3	0	1	1
1	1	1	4	1	1,00	1,00	6	3	0	4	0	0	1
2	2	1	5	3	0,00	2,00	6	3	0	3	0	1	1
1	2	2	3	4	#NULL!	3,00	6	2	0	3	0	1	1
2	1	2	4	1	1,00	1,00	4	3	0	2	0	0	0
1	1	1	4	1	1,00	1,00	4	1	1	2	0	0	0
1	2	2	4	3	0,00	2,00	5	4	0	1	0	0	1
1	1	1	4	1	1,00	1,00	6	2	0	4	0	0	1
1	1	1	4	3	0,00	2,00	6	1	0	2	0	0	0
1	2	1	4	3	0,00	2,00	3	2	0	4	0	0	0
2	1	2	5	1	1,00	1,00	1	4	0	2	0	0	0
2	1	2	4	3	0,00	2,00	3	3	0	4	0	0	1
1	2	2	4	3	0,00	2,00	3	2	0	4	0	0	1
5	1	2	0	1	1,00	1,00	6	3	0	3	0	0	1
3	1	2	3	1	1,00	1,00	3	2	1	2	0	1	1
3	2	2	3	1	1,00	1,00	5	2	1	3	0	0	1
2	2	1	4	4	#NULL!	3,00	3	3	0	3	0	0	0
1	2	2	3	1	1,00	1,00	6	2	0	3	0	0	1
1	1	1	4	1	1,00	1,00	4	1	0	4	0	0	1
1	1	2	3	1	1,00	1,00	2	2	0	4	0	0	0
1	1	1	4	1	1,00	1,00	5	2	0	3	0	0	0
5	2	2	2	4	#NULL!	3,00	2	3	0	2	0	0	0
1	1	1	2	3	0,00	2,00	3	2	0	4	0	0	0
1	2	1	2	1	1,00	1,00	2	4	0	3	0	0	1
1	1	1	4	5	#NULL!	#NULL!	1	3	0	4	0	0	0
1	2	1	3	3	0,00	2,00	1	2	0	4	0	1	1
1	2	1	3	1	1,00	1,00	1	3	0	2	0	0	1
1	2	1	4	1	1,00	1,00	2	3	0	3	0	0	1
1	1	2	3	1	1,00	1,00	3	2	0	4	0	0	0

1	2	1	4	1	1,00	1,00	1	2	0	4	0	1	1
1	1	1	4	3	0,00	2,00	5	3	0	3	0	0	0
1	2	1	5	3	0,00	2,00	6	3	0	3	0	0	1
1	2	1	4	1	1,00	1,00	1	2	0	4	0	0	1
1	1	2	4	3	0,00	2,00	4	2	0	4	0	0	1
1	2	3	6	1	1,00	1,00	4	2	0	3	0	0	0
1	1	1	4	3	0,00	2,00	6	2	0	4	0	0	0
1	2	1	2	1	1,00	1,00	6	2	0	3	0	0	0
1	1	1	2	3	0,00	2,00	4	3	0	4	0	0	1

CovidFami	CovidFami	SM	MM	HCW	FFN	MedExp	VH_DIC	informatio	efficacy_o	DisSev3	DisSev2	DiseaseSe	SC0	Platform
1	0	1	1	#NULL!	#NULL!	2,00	1,00	3	3	3,00	2	3	53,00	0,00
0	0	#NULL!	1	1	#NULL!	2,00	1,00	1	1	1,00	1	5	44,00	0,00
1	0	#NULL!	1	1	1	3,00	1,00	5	1	3,00	4	3	54,00	0,00
0	0	#NULL!	1	#NULL!	#NULL!	1,00	1,00	5	4	2,00	3	4	64,00	0,00
0	0	#NULL!	#NULL!	#NULL!	1	1,00	1,00	4	3	3,00	4	3	51,00	0,00
0	0	#NULL!	#NULL!	#NULL!	1	1,00	0,00	1	2	5,00	4	1	43,00	0,00
0	0	1	#NULL!	#NULL!	#NULL!	1,00	0,00	1	2	5,00	4	1	40,00	0,00
0	0	1	1	#NULL!	#NULL!	2,00	1,00	1	4	2,00	2	4	51,00	0,00
0	1	1	#NULL!	#NULL!	#NULL!	1,00	1,00	2	3	4,00	2	2	45,00	0,00
0	0	1	1	#NULL!	#NULL!	2,00	1,00	1	4	5,00	5	1	47,00	0,00
0	0	#NULL!	1	#NULL!	#NULL!	1,00	1,00	5	5	4,00	5	2	63,00	0,00
0	0	1	1	#NULL!	1	3,00	1,00	4	5	5,00	5	1	50,00	0,00
0	0	1	1	#NULL!	#NULL!	2,00	0,00	2	5	2,00	4	4	62,00	0,00
0	0	#NULL!	1	#NULL!	#NULL!	1,00	1,00	5	3	2,00	4	4	59,00	0,00
1	0	#NULL!	1	#NULL!	1	2,00	0,00	5	3	3,00	4	3	55,00	0,00
0	1	#NULL!	#NULL!	1	#NULL!	1,00	0,00	4	3	3,00	3	3	61,00	0,00
1	0	1	1	#NULL!	#NULL!	2,00	0,00	4	5	4,00	5	2	55,00	0,00
0	0	1	1	1	#NULL!	3,00	0,00	1	3	3,00	4	3	66,00	0,00
0	0	1	1	1	#NULL!	3,00	1,00	1	2	3,00	4	3	60,00	0,00
0	0	#NULL!	1	#NULL!	#NULL!	1,00	1,00	1	1	5,00	1	1	39,00	0,00
1	0	#NULL!	#NULL!	1	#NULL!	1,00	0,00	3	3	2,00	1	4	56,00	0,00
0	0	#NULL!	#NULL!	#NULL!	1	1,00	0,00	5	5	3,00	4	3	69,00	0,00
0	0	#NULL!	1	#NULL!	1	2,00	1,00	4	2	5,00	2	1	43,00	0,00
0	0	#NULL!	#NULL!	1	#NULL!	1,00	0,00	2	5	5,00	5	1	48,00	0,00
0	0	#NULL!	1	#NULL!	#NULL!	1,00	1,00	2	5	5,00	5	1	48,00	0,00
0	0	1	#NULL!	#NULL!	#NULL!	1,00	0,00	1	5	2,00	1	4	41,00	0,00
1	0	1	1	#NULL!	#NULL!	2,00	0,00	4	2	3,00	5	3	53,00	0,00
0	0	#NULL!	#NULL!	1	#NULL!	1,00	0,00	1	2	4,00	2	2	48,00	0,00
0	0	1	#NULL!	#NULL!	#NULL!	1,00	0,00	4	4	4,00	3	2	51,00	0,00
1	0	#NULL!	1	1	#NULL!	2,00	1,00	4	4	3,00	4	3	58,00	0,00
0	0	1	#NULL!	#NULL!	#NULL!	1,00	0,00	2	4	4,00	4	2	49,00	0,00
0	0	#NULL!	1	1	1	3,00	1,00	4	1	1,00	1	5	63,00	0,00

0	0	1	#NULL!	#NULL!	#NULL!	1,00	1,00	4	4	1,00	1	5	52,00	0,00
0	0	1	1	1	#NULL!	3,00	1,00	1	1	5,00	3	1	38,00	0,00
0	0	#NULL!	1	#NULL!	#NULL!	1,00	0,00	1	5	5,00	3	1	45,00	0,00
0	0	#NULL!	1	1	1	3,00	0,00	2	4	3,00	1	3	51,00	0,00
0	0	1	1	#NULL!	#NULL!	2,00	1,00	1	4	4,00	4	2	42,00	0,00
1	0	1	1	#NULL!	1	3,00	1,00	1	5	3,00	4	3	52,00	0,00
1	1	#NULL!	1	#NULL!	#NULL!	1,00	1,00	5	3	5,00	1	1	60,00	0,00
0	0	#NULL!	1	#NULL!	#NULL!	1,00	1,00	2	4	4,00	4	2	53,00	0,00
0	0	#NULL!	1	#NULL!	#NULL!	1,00	0,00	4	5	4,00	2	2	57,00	0,00
0	0	#NULL!	1	1	1	3,00	1,00	4	1	1,00	1	5	58,00	0,00
0	0	#NULL!	1	1	1	3,00	1,00	2	5	5,00	1	1	56,00	0,00
0	0	#NULL!	1	1	1	3,00	1,00	2	5	1,00	4	5	69,00	0,00
0	0	#NULL!	1	1	1	3,00	1,00	2	5	5,00	3	1	56,00	0,00
0	0	1	#NULL!	#NULL!	#NULL!	1,00	0,00	5	4	5,00	4	1	55,00	0,00
0	0	#NULL!	1	1	#NULL!	2,00	0,00	1	4	3,00	4	3	51,00	0,00
0	0	#NULL!	1	#NULL!	#NULL!	1,00	1,00	2	4	4,00	3	2	60,00	0,00
0	0	1	1	#NULL!	#NULL!	2,00	1,00	1	4	4,00	4	2	56,00	0,00
0	0	1	1	#NULL!	#NULL!	2,00	1,00	4	2	5,00	3	1	50,00	0,00
0	0	1	#NULL!	#NULL!	#NULL!	1,00	0,00	5	4	3,00	2	3	57,00	0,00
0	0	1	1	#NULL!	#NULL!	2,00	0,00	3	4	2,00	3	4	58,00	0,00
0	0	#NULL!	1	#NULL!	1	2,00	0,00	4	4	2,00	3	4	62,00	0,00
0	0	#NULL!	1	1	1	3,00	0,00	4	4	4,00	2	2	66,00	0,00
0	0	#NULL!	1	#NULL!	1	2,00	0,00	4	4	2,00	3	4	63,00	0,00
1	0	1	#NULL!	#NULL!	#NULL!	1,00	1,00	1	4	5,00	5	1	52,00	0,00
0	0	#NULL!	1	#NULL!	#NULL!	1,00	0,00	1	2	5,00	4	1	43,00	0,00
1	0	#NULL!	1	#NULL!	#NULL!	1,00	1,00	5	2	3,00	3	3	59,00	0,00
0	0	1	#NULL!	#NULL!	#NULL!	1,00	0,00	1	5	2,00	4	4	54,00	0,00
0	0	#NULL!	1	#NULL!	#NULL!	1,00	0,00	2	5	5,00	5	1	46,00	0,00
0	0	#NULL!	1	#NULL!	#NULL!	1,00	1,00	2	3	4,00	2	2	43,00	0,00
1	0	#NULL!	#NULL!	1	#NULL!	1,00	1,00	1	5	5,00	1	1	43,00	0,00
1	0	#NULL!	#NULL!	1	#NULL!	1,00	1,00	2	4	4,00	3	2	53,00	0,00
0	0	1	1	#NULL!	#NULL!	2,00	1,00	2	3	5,00	5	1	60,00	0,00
0	0	#NULL!	1	#NULL!	#NULL!	1,00	0,00	1	5	5,00	4	1	43,00	0,00

1	0	#NULL!	1	#NULL!	#NULL!	1,00	1,00	3	3	1,00	5	5	61,00	0,00
0	0	#NULL!	1	#NULL!	#NULL!	1,00	0,00	1	5	5,00	5	1	44,00	0,00
0	0	#NULL!	1	#NULL!	#NULL!	1,00	0,00	4	4	3,00	4	3	68,00	0,00
0	0	#NULL!	#NULL!	1	#NULL!	1,00	0,00	5	1	5,00	1	1	52,00	0,00
0	0	1	1	#NULL!	#NULL!	2,00	1,00	4	4	2,00	3	4	53,00	0,00
0	0	#NULL!	1	#NULL!	#NULL!	1,00	1,00	1	3	5,00	5	1	49,00	0,00
0	0	#NULL!	1	#NULL!	#NULL!	1,00	1,00	5	5	3,00	5	3	66,00	0,00
1	0	1	#NULL!	1	#NULL!	2,00	0,00	2	5	5,00	1	1	47,00	0,00
0	0	#NULL!	1	#NULL!	#NULL!	1,00	1,00	2	5	1,00	1	5	50,00	0,00
0	0	#NULL!	#NULL!	#NULL!	1	1,00	0,00	4	4	1,00	2	5	58,00	0,00
0	0	#NULL!	1	#NULL!	#NULL!	1,00	0,00	4	3	3,00	5	3	63,00	0,00
0	0	#NULL!	1	#NULL!	#NULL!	1,00	0,00	5	4	5,00	5	1	50,00	0,00
0	0	#NULL!	1	#NULL!	#NULL!	1,00	1,00	4	2	3,00	5	3	63,00	0,00
0	0	#NULL!	1	1	#NULL!	2,00	0,00	4	2	3,00	4	3	65,00	0,00
1	0	1	#NULL!	#NULL!	#NULL!	1,00	0,00	4	3	3,00	5	3	56,00	0,00
0	0	#NULL!	#NULL!	#NULL!	1	1,00	1,00	4	3	2,00	5	4	71,00	0,00
0	0	#NULL!	1	#NULL!	#NULL!	1,00	0,00	5	5	1,00	4	5	69,00	0,00
0	0	#NULL!	1	#NULL!	#NULL!	1,00	0,00	5	5	5,00	5	1	55,00	0,00
0	0	#NULL!	1	#NULL!	#NULL!	1,00	0,00	5	5	3,00	3	3	62,00	0,00
0	0	#NULL!	1	#NULL!	#NULL!	1,00	0,00	5	5	5,00	5	1	62,00	0,00
0	0	#NULL!	1	#NULL!	#NULL!	1,00	0,00	5	5	1,00	2	5	59,00	0,00
0	0	1	#NULL!	#NULL!	#NULL!	1,00	1,00	4	4	4,00	4	2	55,00	0,00
0	0	#NULL!	1	#NULL!	#NULL!	1,00	0,00	3	4	1,00	5	5	68,00	0,00
0	0	#NULL!	1	#NULL!	#NULL!	1,00	1,00	4	4	3,00	3	3	56,00	0,00
0	0	#NULL!	#NULL!	#NULL!	1	1,00	0,00	4	4	2,00	5	4	68,00	0,00
0	0	#NULL!	1	#NULL!	#NULL!	1,00	1,00	4	4	2,00	4	4	60,00	0,00
0	0	#NULL!	1	1	1	3,00	1,00	4	2	3,00	2	3	51,00	0,00
0	0	1	#NULL!	#NULL!	#NULL!	1,00	1,00	1	3	3,00	3	3	56,00	0,00
0	0	#NULL!	1	#NULL!	#NULL!	1,00	1,00	3	4	1,00	4	5	57,00	0,00
0	0	#NULL!	1	#NULL!	#NULL!	1,00	1,00	5	5	5,00	5	1	74,00	0,00
0	0	#NULL!	1	#NULL!	#NULL!	1,00	1,00	1	5	2,00	5	4	64,00	0,00
0	0	#NULL!	1	#NULL!	#NULL!	1,00	0,00	4	5	3,00	4	3	65,00	0,00
0	0	#NULL!	1	#NULL!	#NULL!	1,00	0,00	1	4	3,00	1	3	52,00	0,00

0	0	#NULL!	1	#NULL!	#NULL!	1,00	1,00	2	5	4,00	2	2	51,00	0,00
0	0	#NULL!	1	#NULL!	#NULL!	1,00	1,00	5	5	1,00	5	5	67,00	0,00
0	0	#NULL!	1	#NULL!	#NULL!	1,00	1,00	3	5	5,00	5	1	57,00	0,00
0	0	1	#NULL!	#NULL!	#NULL!	1,00	1,00	1	3	3,00	1	3	45,00	0,00
1	0	#NULL!	1	#NULL!	#NULL!	1,00	1,00	4	4	3,00	4	3	61,00	0,00
0	0	#NULL!	1	#NULL!	#NULL!	1,00	0,00	5	5	5,00	5	1	57,00	0,00
0	0	#NULL!	1	#NULL!	#NULL!	1,00	1,00	5	2	5,00	5	1	58,00	0,00
0	0	#NULL!	#NULL!	1	#NULL!	1,00	0,00	4	2	3,00	3	3	58,00	0,00
0	0	#NULL!	1	#NULL!	#NULL!	1,00	1,00	1	3	4,00	5	2	63,00	0,00
1	0	#NULL!	1	1	1	3,00	1,00	1	2	1,00	3	5	58,00	0,00
0	0	#NULL!	1	#NULL!	#NULL!	1,00	0,00	3	3	1,00	5	5	58,00	0,00
0	0	#NULL!	1	#NULL!	#NULL!	1,00	1,00	5	5	5,00	2	1	45,00	0,00
0	0	#NULL!	1	#NULL!	#NULL!	1,00	1,00	5	4	2,00	4	4	64,00	0,00
0	0	#NULL!	1	#NULL!	#NULL!	1,00	0,00	2	5	4,00	2	2	53,00	0,00
0	0	1	#NULL!	#NULL!	#NULL!	1,00	1,00	1	3	1,00	5	5	55,00	0,00
0	0	#NULL!	1	#NULL!	#NULL!	1,00	1,00	2	4	5,00	5	1	47,00	0,00
0	0	#NULL!	1	1	#NULL!	2,00	0,00	4	4	2,00	5	4	63,00	0,00
0	0	#NULL!	1	#NULL!	#NULL!	1,00	1,00	4	4	3,00	3	3	52,00	0,00
0	0	#NULL!	#NULL!	1	#NULL!	1,00	0,00	4	5	1,00	1	5	64,00	0,00
0	0	#NULL!	1	#NULL!	#NULL!	1,00	1,00	1	1	3,00	1	3	39,00	0,00
0	0	#NULL!	1	#NULL!	#NULL!	1,00	1,00	1	5	5,00	3	1	50,00	0,00
0	0	#NULL!	1	1	#NULL!	2,00	1,00	5	3	5,00	4	1	58,00	0,00
0	0	#NULL!	1	#NULL!	#NULL!	1,00	1,00	1	1	5,00	1	1	31,00	0,00
0	0	#NULL!	1	#NULL!	#NULL!	1,00	0,00	3	2	3,00	5	3	47,00	0,00
0	0	#NULL!	1	#NULL!	#NULL!	1,00	1,00	2	5	3,00	3	3	57,00	0,00
0	0	1	1	#NULL!	#NULL!	2,00	1,00	4	4	5,00	4	1	52,00	0,00
0	0	#NULL!	1	#NULL!	#NULL!	1,00	1,00	5	3	3,00	5	3	57,00	0,00
1	1	#NULL!	1	#NULL!	#NULL!	1,00	0,00	5	5	4,00	2	2	51,00	0,00
1	0	#NULL!	#NULL!	#NULL!	1	1,00	1,00	1	4	5,00	4	1	54,00	0,00
0	0	#NULL!	1	#NULL!	#NULL!	1,00	0,00	3	5	3,00	2	3	52,00	0,00
1	1	#NULL!	1	#NULL!	#NULL!	1,00	0,00	1	5	3,00	2	3	46,00	0,00
0	0	#NULL!	1	1	#NULL!	2,00	1,00	1	1	5,00	1	1	35,00	0,00
0	0	#NULL!	1	#NULL!	1	2,00	1,00	5	5	1,00	5	5	60,00	0,00

1	0	1	#NULL!	#NULL!	#NULL!	1,00	1,00	2	1	5,00	5	1	58,00	0,00
0	0	1	1	#NULL!	#NULL!	2,00	0,00	4	4	2,00	1	4	61,00	0,00
0	0	#NULL!	1	#NULL!	#NULL!	1,00	1,00	3	1	1,00	1	5	49,00	0,00
0	0	1	1	#NULL!	1	3,00	1,00	1	5	2,00	5	4	48,00	0,00
0	0	#NULL!	#NULL!	#NULL!	1	1,00	0,00	5	5	3,00	3	3	69,00	0,00
0	0	#NULL!	1	#NULL!	#NULL!	1,00	0,00	4	5	1,00	5	5	78,00	0,00
0	0	#NULL!	1	#NULL!	#NULL!	1,00	1,00	5	3	1,00	4	5	54,00	0,00
0	0	#NULL!	1	#NULL!	#NULL!	1,00	0,00	2	5	2,00	4	4	57,00	0,00
0	0	#NULL!	1	#NULL!	#NULL!	1,00	1,00	2	4	4,00	2	2	48,00	0,00
0	1	#NULL!	1	#NULL!	#NULL!	1,00	0,00	1	5	3,00	4	3	52,00	0,00
0	0	1	1	#NULL!	#NULL!	1,00	0,00	1	4	2,00	1	4	43,00	0,00
0	0	#NULL!	1	1	1	3,00	0,00	1	3	4,00	4	2	53,00	0,00
0	0	1	#NULL!	#NULL!	#NULL!	1,00	0,00	4	4	5,00	5	1	58,00	0,00
1	0	1	1	#NULL!	#NULL!	2,00	1,00	1	1	1,00	1	5	53,00	0,00
0	0	1	1	#NULL!	#NULL!	2,00	1,00	2	4	1,00	5	5	56,00	0,00
0	0	#NULL!	1	1	1	3,00	1,00	4	2	3,00	4	3	57,00	0,00
0	0	#NULL!	1	#NULL!	#NULL!	1,00	1,00	1	3	3,00	5	3	58,00	0,00
0	0	1	1	#NULL!	#NULL!	2,00	0,00	2	4	2,00	5	4	59,00	0,00
0	0	#NULL!	1	#NULL!	#NULL!	1,00	1,00	2	4	2,00	4	4	64,00	0,00
0	0	#NULL!	1	#NULL!	#NULL!	1,00	1,00	2	4	4,00	5	2	54,00	0,00
0	0	1	1	#NULL!	#NULL!	2,00	1,00	2	3	3,00	2	3	50,00	0,00
0	0	#NULL!	1	#NULL!	#NULL!	1,00	1,00	5	1	1,00	5	5	65,00	0,00
0	0	#NULL!	#NULL!	1	#NULL!	1,00	1,00	5	5	1,00	5	5	78,00	0,00
0	0	#NULL!	#NULL!	#NULL!	1	1,00	1,00	3	1	3,00	5	3	55,00	0,00
0	0	#NULL!	1	#NULL!	1	2,00	1,00	2	3	5,00	1	1	42,00	0,00
1	1	1	1	#NULL!	1	3,00	1,00	4	4	1,00	1	5	53,00	0,00
0	0	#NULL!	1	1	1	3,00	0,00	4	4	3,00	5	3	65,00	0,00
0	0	#NULL!	1	1	1	3,00	1,00	5	5	2,00	4	4	63,00	0,00
0	0	1	#NULL!	#NULL!	1	2,00	1,00	3	3	3,00	5	3	63,00	0,00
0	0	#NULL!	1	#NULL!	#NULL!	1,00	0,00	2	5	5,00	5	1	51,00	0,00
0	0	1	#NULL!	#NULL!	#NULL!	1,00	0,00	1	4	5,00	5	1	43,00	0,00
0	0	1	1	#NULL!	#NULL!	2,00	0,00	1	5	1,00	1	5	44,00	0,00
1	0	#NULL!	1	1	#NULL!	2,00	1,00	1	5	3,00	3	3	54,00	0,00

0	0	1	#NULL!	#NULL!	#NULL!	1,00	1,00	1	5	1,00	3	5	57,00	0,00
1	0	1	1	#NULL!	#NULL!	2,00	1,00	2	4	3,00	5	3	59,00	0,00
1	0	#NULL!	1	1	#NULL!	2,00	1,00	4	3	3,00	2	3	58,00	0,00
0	0	1	#NULL!	#NULL!	#NULL!	1,00	0,00	1	5	4,00	4	2	52,00	0,00
1	1	1	#NULL!	#NULL!	#NULL!	1,00	1,00	4	5	3,00	5	3	59,00	0,00
0	0	#NULL!	1	#NULL!	#NULL!	1,00	1,00	4	4	3,00	4	3	54,00	0,00
1	1	#NULL!	1	#NULL!	#NULL!	1,00	1,00	4	4	4,00	4	2	52,00	0,00
0	0	1	1	#NULL!	#NULL!	2,00	0,00	4	4	2,00	5	4	73,00	0,00
0	0	#NULL!	#NULL!	1	#NULL!	1,00	1,00	3	2	1,00	3	5	58,00	0,00
1	0	1	#NULL!	#NULL!	#NULL!	1,00	1,00	4	3	4,00	4	2	53,00	0,00
0	0	#NULL!	1	#NULL!	#NULL!	1,00	0,00	4	5	2,00	5	4	79,00	0,00
0	0	#NULL!	1	#NULL!	#NULL!	1,00	1,00	4	3	2,00	4	4	60,00	0,00
1	0	1	1	#NULL!	1	3,00	0,00	1	4	2,00	4	4	59,00	0,00
0	0	#NULL!	1	1	1	3,00	0,00	5	2	1,00	5	5	72,00	0,00
0	0	#NULL!	1	#NULL!	#NULL!	1,00	1,00	2	4	5,00	5	1	55,00	0,00
1	1	1	#NULL!	#NULL!	#NULL!	1,00	1,00	4	2	2,00	4	4	61,00	0,00
0	0	1	1	#NULL!	#NULL!	2,00	0,00	4	5	2,00	4	4	57,00	0,00
1	0	1	#NULL!	#NULL!	#NULL!	1,00	0,00	4	1	3,00	3	3	63,00	0,00
1	0	1	#NULL!	#NULL!	#NULL!	1,00	1,00	3	4	4,00	1	2	54,00	0,00
0	0	#NULL!	1	#NULL!	1	2,00	1,00	2	1	5,00	2	1	51,00	0,00
1	0	1	1	#NULL!	1	3,00	0,00	4	4	5,00	2	1	57,00	0,00
1	1	1	1	#NULL!	#NULL!	2,00	0,00	4	4	5,00	5	1	53,00	0,00
0	0	#NULL!	1	#NULL!	#NULL!	1,00	0,00	4	5	1,00	5	5	56,00	0,00
0	0	#NULL!	1	#NULL!	#NULL!	1,00	0,00	5	1	5,00	2	1	44,00	0,00
0	0	#NULL!	1	#NULL!	#NULL!	1,00	0,00	5	1	5,00	2	1	42,00	0,00
0	0	#NULL!	1	#NULL!	#NULL!	1,00	1,00	1	5	4,00	4	2	49,00	0,00
1	0	1	1	#NULL!	#NULL!	2,00	1,00	5	5	5,00	5	1	50,00	0,00
0	0	1	1	#NULL!	1	3,00	1,00	2	2	4,00	1	2	51,00	0,00
0	0	#NULL!	1	#NULL!	#NULL!	1,00	0,00	1	4	5,00	3	1	46,00	0,00
0	0	#NULL!	1	#NULL!	#NULL!	1,00	0,00	5	5	2,00	5	4	76,00	0,00
0	0	#NULL!	1	#NULL!	#NULL!	1,00	1,00	1	1	2,00	5	4	71,00	0,00
1	0	#NULL!	1	#NULL!	1	2,00	1,00	5	2	1,00	1	5	56,00	0,00
0	0	1	#NULL!	#NULL!	#NULL!	1,00	1,00	4	4	3,00	4	3	52,00	0,00

0	0	#NULL!	1	#NULL!	#NULL!	1,00	1,00	1	3	1,00	1	5	47,00	0,00
0	0	#NULL!	1	#NULL!	#NULL!	1,00	1,00	5	4	3,00	1	3	65,00	0,00
0	0	1	#NULL!	#NULL!	#NULL!	1,00	1,00	2	4	3,00	2	3	46,00	0,00
1	0	1	1	#NULL!	#NULL!	2,00	1,00	2	1	3,00	1	3	44,00	0,00
0	0	#NULL!	1	#NULL!	#NULL!	1,00	0,00	1	5	5,00	5	1	48,00	0,00
0	0	1	1	#NULL!	1	3,00	1,00	1	5	5,00	1	1	47,00	0,00
0	0	1	1	#NULL!	#NULL!	2,00	0,00	3	5	2,00	1	4	47,00	0,00
0	0	#NULL!	1	#NULL!	#NULL!	1,00	0,00	4	5	3,00	5	3	60,00	0,00
0	0	#NULL!	1	1	#NULL!	2,00	1,00	4	3	1,00	1	5	49,00	0,00
0	0	#NULL!	1	#NULL!	#NULL!	1,00	1,00	1	1	3,00	1	3	56,00	0,00
0	0	#NULL!	1	#NULL!	#NULL!	1,00	1,00	1	1	3,00	1	3	48,00	0,00
0	0	1	1	#NULL!	#NULL!	2,00	0,00	3	2	5,00	5	1	49,00	0,00
0	0	1	#NULL!	#NULL!	#NULL!	1,00	1,00	1	4	1,00	1	5	48,00	0,00
0	0	1	#NULL!	#NULL!	#NULL!	1,00	1,00	2	1	3,00	1	3	44,00	0,00
0	0	#NULL!	1	#NULL!	#NULL!	1,00	1,00	1	1	5,00	5	1	48,00	0,00
0	0	#NULL!	1	#NULL!	1	2,00	1,00	4	5	1,00	5	5	67,00	0,00
0	0	1	#NULL!	#NULL!	#NULL!	1,00	1,00	5	5	5,00	4	1	52,00	0,00
0	0	#NULL!	1	#NULL!	#NULL!	1,00	0,00	1	5	5,00	4	1	42,00	0,00
0	0	1	1	#NULL!	#NULL!	2,00	0,00	4	5	1,00	5	5	65,00	0,00
0	0	1	1	#NULL!	#NULL!	2,00	0,00	4	5	1,00	5	5	70,00	0,00
0	0	#NULL!	1	#NULL!	#NULL!	1,00	1,00	5	5	1,00	2	5	73,00	0,00
0	0	#NULL!	1	1	#NULL!	2,00	1,00	4	5	5,00	5	1	51,00	0,00
0	0	#NULL!	#NULL!	#NULL!	1	1,00	0,00	4	2	3,00	4	3	62,00	0,00
0	0	#NULL!	1	#NULL!	#NULL!	1,00	0,00	4	4	5,00	5	1	51,00	0,00
0	0	#NULL!	1	#NULL!	#NULL!	1,00	0,00	3	4	5,00	4	1	49,00	0,00
0	0	#NULL!	1	#NULL!	#NULL!	1,00	0,00	2	4	5,00	5	1	64,00	0,00
0	0	#NULL!	#NULL!	1	1	2,00	0,00	1	5	5,00	1	1	47,00	0,00
1	1	#NULL!	1	#NULL!	#NULL!	1,00	1,00	2	5	1,00	5	5	66,00	0,00
0	0	#NULL!	1	#NULL!	#NULL!	1,00	1,00	2	5	5,00	5	1	45,00	0,00
1	0	#NULL!	1	#NULL!	#NULL!	1,00	0,00	4	5	3,00	5	3	62,00	0,00
0	0	#NULL!	1	#NULL!	#NULL!	1,00	1,00	2	4	3,00	3	3	61,00	0,00
0	0	#NULL!	1	#NULL!	#NULL!	1,00	0,00	1	4	5,00	1	1	54,00	0,00
0	0	1	#NULL!	#NULL!	#NULL!	1,00	0,00	1	4	5,00	2	1	55,00	0,00

0	0	1	#NULL!	#NULL!	#NULL!	1,00	0,00	1	5	4,00	4	2	50,00	0,00
0	0	#NULL!	1	#NULL!	#NULL!	1,00	1,00	5	4	4,00	2	2	64,00	0,00
1	0	1	#NULL!	#NULL!	#NULL!	1,00	0,00	4	4	4,00	4	2	45,00	0,00
0	0	#NULL!	1	#NULL!	1	2,00	0,00	2	2	3,00	3	3	62,00	0,00
1	0	1	#NULL!	#NULL!	#NULL!	1,00	1,00	1	4	3,00	2	3	46,00	0,00
0	0	1	1	1	#NULL!	3,00	1,00	1	1	1,00	1	5	49,00	0,00
0	0	1	#NULL!	#NULL!	#NULL!	1,00	1,00	1	3	3,00	4	3	49,00	0,00
0	0	1	1	#NULL!	#NULL!	2,00	1,00	2	4	5,00	2	1	#NULL!	1,00
0	0	1	1	#NULL!	#NULL!	2,00	1,00	1	5	5,00	1	1	#NULL!	1,00
0	0	#NULL!	#NULL!	1	#NULL!	1,00	0,00	4	2	4,00	3	2	#NULL!	1,00
0	0	#NULL!	1	#NULL!	#NULL!	1,00	1,00	2	2	4,00	4	2	#NULL!	1,00
1	1	1	1	#NULL!	#NULL!	2,00	1,00	2	4	3,00	4	3	#NULL!	1,00
0	0	1	1	#NULL!	#NULL!	2,00	0,00	1	3	5,00	4	1	#NULL!	1,00
0	0	1	#NULL!	#NULL!	#NULL!	1,00	1,00	3	4	5,00	2	1	#NULL!	1,00
0	0	1	1	#NULL!	#NULL!	2,00	0,00	1	5	5,00	4	1	#NULL!	1,00
0	0	1	#NULL!	#NULL!	#NULL!	1,00	1,00	2	1	5,00	2	1	#NULL!	1,00
1	0	#NULL!	#NULL!	1	#NULL!	1,00	1,00	1	3	5,00	1	1	#NULL!	1,00
0	0	1	1	1	1	4,00	1,00	5	2	2,00	3	4	64,00	0,00
0	0	1	1	1	1	4,00	1,00	1	3	3,00	5	3	62,00	0,00
0	1	1	1	1	1	4,00	1,00	5	3	1,00	1	5	59,00	0,00
1	0	1	1	1	1	4,00	0,00	5	5	2,00	5	4	72,00	0,00
0	0	1	1	1	1	4,00	1,00	1	4	3,00	4	3	62,00	0,00
1	0	1	1	1	1	4,00	0,00	1	5	1,00	5	5	74,00	0,00
0	0	1	1	1	1	4,00	1,00	1	4	5,00	2	1	46,00	0,00
1	0	1	1	1	1	4,00	0,00	4	4	2,00	3	4	68,00	0,00
0	0	1	1	1	1	4,00	0,00	1	5	4,00	1	2	47,00	0,00
1	0	1	1	1	1	4,00	1,00	2	1	2,00	1	4	53,00	0,00
0	0	1	1	1	1	4,00	1,00	1	2	5,00	5	1	52,00	0,00
0	0	1	1	1	1	4,00	1,00	4	3	2,00	1	4	51,00	0,00
0	0	1	1	1	1	4,00	1,00	1	4	5,00	5	1	50,00	0,00
0	0	1	1	1	1	4,00	0,00	2	5	4,00	5	2	49,00	0,00
0	0	1	1	1	1	4,00	0,00	4	5	4,00	3	2	69,00	0,00
0	0	1	1	1	1	4,00	0,00	4	5	5,00	2	1	64,00	0,00

1	0	1	1	1	1	4,00	1,00	2	1	1,00	3	5	57,00	0,00
1	1	1	1	1	1	4,00	1,00	1	5	1,00	5	5	68,00	0,00
0	1	1	1	1	1	4,00	0,00	4	3	5,00	3	1	59,00	0,00
0	0	1	1	1	1	4,00	1,00	4	1	2,00	4	4	61,00	0,00
1	0	1	1	1	1	4,00	0,00	4	4	4,00	4	2	58,00	0,00
0	0	1	1	1	1	4,00	0,00	3	4	2,00	4	4	70,00	0,00
0	0	1	1	1	1	4,00	0,00	2	5	5,00	5	1	56,00	0,00
0	0	1	1	1	1	4,00	0,00	5	2	3,00	5	3	65,00	0,00
0	1	1	1	1	1	4,00	1,00	1	4	5,00	3	1	53,00	0,00
0	0	1	1	1	1	4,00	1,00	5	1	1,00	1	5	49,00	0,00
0	0	1	1	1	1	4,00	0,00	1	3	3,00	3	3	53,00	0,00
1	0	1	1	1	1	4,00	0,00	1	5	5,00	4	1	51,00	0,00
0	0	1	1	1	1	4,00	0,00	1	5	5,00	5	1	#NULL!	1,00
0	0	1	1	#NULL!	1	3,00	0,00	1	4	2,00	4	4	52,00	0,00
0	0	1	1	#NULL!	#NULL!	2,00	1,00	3	2	5,00	2	1	38,00	0,00
0	0	#NULL!	1	#NULL!	#NULL!	1,00	1,00	4	3	2,00	4	4	60,00	0,00
1	0	#NULL!	1	#NULL!	#NULL!	1,00	0,00	1	3	2,00	5	4	58,00	0,00
0	0	#NULL!	1	#NULL!	#NULL!	1,00	1,00	5	2	4,00	5	2	49,00	0,00
0	0	#NULL!	1	#NULL!	#NULL!	1,00	1,00	5	5	1,00	3	5	63,00	0,00
0	0	1	1	#NULL!	#NULL!	2,00	0,00	1	5	2,00	1	4	43,00	0,00
0	0	1	#NULL!	#NULL!	#NULL!	1,00	0,00	2	4	3,00	4	3	50,00	0,00
1	0	#NULL!	1	#NULL!	#NULL!	1,00	0,00	4	4	3,00	4	3	54,00	0,00
1	0	#NULL!	1	#NULL!	#NULL!	1,00	0,00	2	5	4,00	4	2	55,00	0,00
0	0	1	1	#NULL!	#NULL!	2,00	0,00	5	2	4,00	5	2	49,00	0,00
0	0	1	1	#NULL!	1	3,00	0,00	1	5	1,00	5	5	50,00	0,00
1	0	1	#NULL!	1	1	3,00	0,00	5	5	4,00	5	2	67,00	0,00
1	1	1	1	#NULL!	1	3,00	0,00	4	5	5,00	5	1	77,00	0,00
1	0	1	#NULL!	#NULL!	#NULL!	1,00	0,00	1	5	5,00	4	1	54,00	0,00
0	0	1	1	#NULL!	#NULL!	2,00	0,00	1	5	4,00	1	2	44,00	0,00
0	0	#NULL!	1	1	#NULL!	2,00	0,00	4	4	2,00	5	4	55,00	0,00
0	1	#NULL!	#NULL!	1	#NULL!	1,00	1,00	2	2	5,00	2	1	46,00	0,00
1	0	1	1	#NULL!	#NULL!	2,00	0,00	5	3	5,00	5	1	56,00	0,00
1	0	#NULL!	1	1	1	3,00	0,00	5	5	5,00	5	1	68,00	0,00

1	0	#NULL!	1	#NULL!	#NULL!	1,00	0,00	1	4	2,00	4	4	66,00	0,00
0	0	1	#NULL!	#NULL!	#NULL!	1,00	0,00	2	2	3,00	4	3	48,00	0,00
0	1	#NULL!	#NULL!	1	#NULL!	1,00	1,00	1	1	4,00	4	2	46,00	0,00
0	0	1	1	#NULL!	#NULL!	2,00	0,00	1	1	2,00	5	4	40,00	0,00
1	0	1	#NULL!	#NULL!	#NULL!	1,00	0,00	1	2	1,00	3	5	49,00	0,00
0	0	#NULL!	1	#NULL!	#NULL!	1,00	0,00	1	5	4,00	5	2	53,00	0,00
1	0	#NULL!	1	#NULL!	#NULL!	1,00	0,00	4	3	2,00	3	4	70,00	0,00
1	0	#NULL!	1	1	#NULL!	2,00	0,00	1	4	5,00	1	1	50,00	0,00
0	0	#NULL!	1	#NULL!	#NULL!	1,00	0,00	2	4	5,00	4	1	62,00	0,00
0	0	#NULL!	1	#NULL!	#NULL!	1,00	1,00	4	5	5,00	5	1	54,00	0,00
0	0	#NULL!	1	1	#NULL!	2,00	0,00	1	4	4,00	5	2	46,00	0,00
0	0	#NULL!	1	#NULL!	#NULL!	1,00	1,00	1	3	5,00	1	1	54,00	0,00
0	0	#NULL!	1	1	1	3,00	0,00	4	4	2,00	5	4	62,00	0,00
0	0	#NULL!	1	#NULL!	#NULL!	1,00	0,00	1	1	5,00	3	1	38,00	0,00
1	0	1	1	#NULL!	#NULL!	2,00	0,00	1	2	4,00	5	2	61,00	0,00
0	0	#NULL!	1	#NULL!	#NULL!	1,00	0,00	1	5	2,00	5	4	65,00	0,00
0	0	#NULL!	1	#NULL!	#NULL!	1,00	1,00	2	3	1,00	5	5	67,00	0,00
0	0	#NULL!	1	#NULL!	#NULL!	1,00	0,00	1	1	5,00	5	1	58,00	0,00
1	0	1	#NULL!	#NULL!	#NULL!	1,00	0,00	5	4	2,00	4	4	69,00	0,00
0	0	#NULL!	1	1	1	3,00	0,00	5	5	2,00	3	4	73,00	0,00
0	0	#NULL!	1	#NULL!	#NULL!	1,00	0,00	4	4	1,00	5	5	57,00	0,00
0	0	#NULL!	1	#NULL!	#NULL!	1,00	0,00	1	4	5,00	1	1	44,00	0,00
0	0	#NULL!	1	#NULL!	#NULL!	1,00	1,00	4	4	2,00	4	4	54,00	0,00
0	0	#NULL!	1	1	#NULL!	2,00	0,00	4	2	4,00	2	2	54,00	0,00
0	0	#NULL!	1	#NULL!	#NULL!	1,00	0,00	2	4	4,00	4	2	50,00	0,00
1	1	#NULL!	1	#NULL!	1	2,00	1,00	4	4	5,00	5	1	68,00	0,00
0	0	#NULL!	#NULL!	1	#NULL!	1,00	0,00	5	5	5,00	5	1	#NULL!	1,00
1	0	#NULL!	#NULL!	1	#NULL!	1,00	0,00	3	4	5,00	4	1	#NULL!	1,00
1	0	1	#NULL!	#NULL!	#NULL!	1,00	1,00	4	4	5,00	4	1	#NULL!	1,00
1	0	1	1	1	1	4,00	0,00	5	5	2,00	1	4	57,00	#NULL!
1	0	1	1	1	1	4,00	0,00	3	3	2,00	4	4	58,00	0,00
1	1	1	1	1	1	4,00	1,00	1	3	1,00	3	5	69,00	0,00
0	0	1	1	1	1	4,00	0,00	1	3	2,00	5	4	56,00	0,00

1	0	1	1	1	1	4,00	0,00	5	4	2,00	5	4	67,00	0,00
1	0	1	1	1	1	4,00	0,00	2	5	3,00	4	3	55,00	0,00
0	0	1	1	1	1	4,00	0,00	4	2	4,00	3	2	70,00	0,00
0	0	1	1	1	1	4,00	0,00	4	5	5,00	1	1	63,00	0,00
1	0	1	1	1	1	4,00	0,00	1	5	2,00	1	4	60,00	0,00
0	0	1	1	1	1	4,00	0,00	4	3	3,00	4	3	66,00	0,00
1	1	1	1	1	1	4,00	0,00	4	4	3,00	4	3	61,00	0,00
1	0	1	1	1	1	4,00	0,00	4	5	5,00	4	1	65,00	0,00
0	0	1	1	1	1	4,00	0,00	5	5	2,00	4	4	#NULL!	1,00

VacHes	VacAtt	ab	effic1	effic2	effic3	religi	cult	safety1	safety2	safety3	sief_ef	perheal3
1	2	0,00	3	3,00	3	1	1	5	5	2	3,60	2,00
1	1	0,00	5	5,00	1	1	5	1	5	1	2,60	3,00
3	1	0,00	3	5,00	1	1	1	2	3	1	2,00	3,00
2	3	0,00	3	2,00	4	3	3	5	5	5	4,40	3,00
2	1	0,00	2	3,00	3	3	3	2	3	3	2,60	3,00
5	1	0,00	2	4,00	2	1	1	2	2	5	2,60	1,00
5	1	0,00	2	4,00	2	1	1	2	2	5	2,60	1,00
2	1	0,00	3	2,00	2	1	1	5	4	5	3,80	3,00
3	2	0,00	1	3,00	2	2	2	2	3	2	2,00	3,00
1	1	0,00	1	2,00	1	4	1	4	1	5	2,40	3,00
1	2	0,00	4	1,00	3	5	4	4	4	5	4,00	3,00
3	1	0,00	3	1,00	1	1	1	3	1	3	2,20	3,00
5	1	0,00	4	1,00	1	1	4	1	4	5	3,00	3,00
1	4	0,00	3	3,00	3	1	3	4	2	3	3,00	3,00
4	3	0,00	3	3,00	3	4	1	1	3	2	2,40	3,00
4	4	0,00	4	3,00	3	2	3	3	3	3	3,20	2,00
4	2	0,00	2	1,00	1	1	1	4	5	5	3,40	3,00
4	1	0,00	4	3,00	5	2	4	4	4	2	3,80	3,00
2	3	0,00	4	4,00	5	2	3	4	4	5	4,40	3,00
1	1	0,00	2	5,00	1	1	1	5	4	3	3,00	3,00
5	4	0,00	1	3,00	2	1	5	2	4	4	2,60	2,00
4	3	0,00	4	1,00	5	3	4	4	4	5	4,40	2,00
2	1	0,00	1	4,00	1	1	2	4	4	5	3,00	1,00
4	1	0,00	1	1,00	1	1	1	3	3	2	2,00	2,00
2	1	0,00	1	1,00	4	1	1	3	3	2	2,60	2,00
4	1	0,00	1	1,00	1	1	1	2	4	5	2,60	3,00
5	1	0,00	3	4,00	1	1	3	4	2	2	2,40	3,00
5	2	0,00	1	4,00	1	4	2	2	1	2	1,40	2,00
5	4	0,00	2	2,00	1	1	1	4	4	4	3,00	2,00
2	4	0,00	4	2,00	3	3	2	3	4	4	3,60	3,00
4	1	0,00	2	2,00	2	1	2	4	3	3	2,80	3,00
1	1	0,00	5	5,00	1	5	1	5	5	5	4,20	3,00

3	1	0,00	3	2,00	1	1	1	4	3	5	3,20	2,00
2	2	0,00	3	5,00	2	2	2	4	1	1	2,20	2,00
5	1	0,00	1	1,00	1	1	1	5	1	4	2,40	3,00
5	1	0,00	1	2,00	4	4	1	4	1	4	2,80	3,00
2	1	0,00	4	2,00	1	1	2	5	2	1	2,60	3,00
3	1	0,00	1	1,00	1	1	1	2	1	4	1,80	3,00
1	1	0,00	3	3,00	5	5	5	5	3	5	4,20	2,00
2	1	0,00	3	2,00	4	4	3	4	4	4	3,80	2,00
5	1	0,00	3	1,00	4	3	3	4	3	4	3,60	2,00
1	2	0,00	3	5,00	1	1	3	3	5	4	3,20	2,00
1	1	0,00	4	1,00	4	4	5	2	4	4	3,60	3,00
1	5	0,00	5	1,00	4	4	5	3	4	4	4,00	1,00
1	1	0,00	2	1,00	1	4	2	5	1	4	2,60	3,00
5	1	0,00	3	2,00	4	2	2	2	2	4	3,00	3,00
4	1	0,00	3	2,00	3	1	1	4	4	4	3,60	2,00
1	1	0,00	5	2,00	4	5	3	5	5	5	4,80	2,00
1	1	0,00	4	2,00	2	5	2	5	4	5	4,00	3,00
1	1	0,00	4	4,00	3	5	1	5	5	5	4,40	3,00
4	2	0,00	4	2,00	2	1	1	5	5	5	4,20	3,00
4	2	0,00	3	2,00	1	4	2	4	4	4	3,20	3,00
4	2	0,00	4	2,00	1	4	2	3	4	4	3,20	3,00
5	2	0,00	2	2,00	4	4	4	4	4	4	3,60	3,00
4	3	0,00	2	2,00	2	4	4	4	4	4	3,20	3,00
2	1	0,00	4	2,00	2	1	1	5	5	5	4,20	3,00
4	1	0,00	1	4,00	1	1	1	4	4	5	3,00	2,00
3	3	0,00	3	4,00	4	2	4	4	3	4	3,60	3,00
5	1	0,00	3	1,00	1	1	1	5	5	4	3,60	3,00
4	1	0,00	1	1,00	1	1	1	4	1	4	2,20	3,00
3	1	0,00	2	3,00	3	1	1	3	3	3	2,80	3,00
3	5	0,00	4	1,00	4	1	1	3	1	4	3,20	1,00
3	2	0,00	3	2,00	3	3	3	3	2	3	2,80	1,00
3	3	0,00	4	3,00	3	4	1	4	3	4	3,60	3,00
5	1	0,00	1	1,00	1	1	1	4	1	2	1,80	3,00

1	1	0,00	3	3,00	4	5	2	3	3	4	3,40	3,00
5	1	0,00	1	1,00	1	1	1	2	2	4	2,00	2,00
4	4	0,00	3	2,00	4	3	4	4	4	3	3,60	2,00
5	4	0,00	3	5,00	5	1	1	1	1	4	2,80	3,00
1	2	0,00	2	2,00	1	1	1	5	3	4	3,00	3,00
3	1	0,00	3	3,00	3	3	3	4	3	5	3,60	2,00
1	1	0,00	3	1,00	5	5	3	5	5	5	4,60	1,00
5	1	0,00	1	1,00	1	1	1	5	3	4	2,80	2,00
1	1	0,00	2	1,00	1	1	1	3	3	1	2,00	3,00
4	1	0,00	3	2,00	4	2	2	4	4	4	3,80	2,00
5	1	0,00	3	3,00	1	3	3	4	4	5	3,40	2,00
4	1	0,00	2	2,00	1	1	1	2	5	5	3,00	3,00
3	2	0,00	3	4,00	3	4	5	5	4	5	4,00	2,00
5	5	0,00	4	4,00	3	5	4	3	5	4	3,80	1,00
4	1	0,00	4	3,00	4	4	1	3	4	4	3,80	3,00
2	4	0,00	4	3,00	3	5	3	5	5	5	4,40	1,00
5	4	0,00	4	1,00	1	3	1	5	5	5	4,00	3,00
5	4	0,00	1	1,00	1	1	1	5	1	5	2,60	3,00
5	2	0,00	5	1,00	4	3	2	3	5	2	3,80	3,00
5	4	0,00	1	1,00	1	1	1	5	1	5	2,60	2,00
5	1	0,00	5	1,00	1	3	1	4	3	5	3,60	3,00
2	3	0,00	3	2,00	3	5	1	4	4	4	3,60	1,00
5	3	0,00	3	2,00	4	3	4	4	5	4	4,00	2,00
2	5	0,00	4	2,00	3	3	4	4	3	4	3,60	1,00
4	3	0,00	4	2,00	3	4	5	4	3	4	3,60	1,00
3	4	0,00	2	2,00	4	4	4	4	4	4	3,60	2,00
2	1	0,00	2	4,00	3	1	2	5	3	2	3,00	3,00
1	4	0,00	3	3,00	3	4	3	3	3	4	3,20	3,00
3	1	0,00	2	2,00	3	2	4	2	4	4	3,00	1,00
2	5	0,00	5	1,00	5	5	5	5	5	5	5,00	3,00
1	1	0,00	1	1,00	5	1	5	5	3	5	3,80	2,00
5	5	0,00	1	1,00	4	5	4	4	3	4	3,20	3,00
5	3	0,00	3	2,00	1	1	4	4	2	1	2,20	3,00

3	3	0,00	3	1,00	2	4	1	2	3	3	2,60	3,00
1	1	0,00	4	1,00	5	5	1	5	5	5	4,80	1,00
1	3	0,00	5	1,00	4	4	2	5	2	4	4,00	2,00
1	4	0,00	3	3,00	4	3	3	3	3	3	3,20	1,00
2	4	0,00	3	2,00	4	1	4	4	4	4	3,80	1,00
5	1	0,00	3	1,00	2	1	1	4	4	4	3,40	3,00
1	1	0,00	5	4,00	5	5	1	5	5	5	5,00	1,00
5	2	0,00	3	4,00	3	3	1	4	5	3	3,60	2,00
3	2	0,00	3	3,00	3	3	3	3	5	3	3,40	3,00
1	1	0,00	4	4,00	1	2	1	4	4	1	2,80	3,00
4	5	0,00	3	3,00	1	5	5	3	3	1	2,20	3,00
2	1	0,00	1	1,00	1	4	1	5	1	5	2,60	2,00
2	3	0,00	4	2,00	4	4	4	5	3	4	4,00	1,00
5	2	0,00	4	1,00	1	2	5	3	4	2	2,80	3,00
1	2	0,00	3	3,00	2	5	2	5	5	2	3,40	2,00
3	1	0,00	1	2,00	1	1	1	4	3	5	2,80	1,00
4	1	0,00	4	2,00	4	4	1	4	4	5	4,20	3,00
3	2	0,00	3	2,00	4	2	3	3	3	2	3,00	3,00
5	1	0,00	1	1,00	1	5	5	2	5	5	2,80	2,00
1	3	0,00	2	5,00	1	4	3	1	1	1	1,20	2,00
1	1	0,00	4	1,00	4	1	4	4	3	4	3,80	2,00
1	1	0,00	3	3,00	4	2	4	5	5	5	4,40	3,00
1	2	0,00	2	5,00	1	2	2	1	2	1	1,40	2,00
5	1	0,00	1	4,00	5	1	1	3	1	1	2,20	3,00
1	3	0,00	4	1,00	4	4	3	1	3	4	3,20	3,00
3	1	0,00	1	2,00	4	2	1	2	4	3	2,80	1,00
1	5	0,00	4	3,00	4	3	5	4	4	1	3,40	1,00
5	1	0,00	1	1,00	1	1	1	4	1	1	1,60	3,00
2	4	0,00	1	2,00	3	3	2	3	4	4	3,00	1,00
5	1	0,00	3	1,00	1	1	1	3	3	3	2,60	3,00
5	1	0,00	1	1,00	1	1	1	4	1	1	1,60	3,00
1	1	0,00	5	5,00	1	1	1	3	1	1	2,20	3,00
1	1	0,00	5	1,00	1	5	1	2	4	5	3,40	3,00

1	1	0,00	4	5,00	1	5	1	5	5	5	4,00	3,00
5	3	0,00	4	2,00	3	3	3	3	1	4	3,00	3,00
1	2	0,00	3	5,00	3	3	3	3	3	4	3,20	2,00
3	1	0,00	3	1,00	1	1	1	3	4	1	2,40	1,00
5	4	0,00	4	1,00	5	3	3	5	3	5	4,40	2,00
5	5	0,00	3	1,00	5	5	5	5	3	1	3,40	1,00
1	1	0,00	1	3,00	1	1	5	5	1	3	2,20	3,00
5	1	0,00	2	1,00	2	4	3	2	2	4	2,40	3,00
1	4	0,00	5	2,00	1	1	1	2	5	4	3,40	1,00
5	5	0,00	1	1,00	1	2	2	3	1	4	2,00	2,00
5	1	0,00	1	2,00	1	4	1	4	2	2	2,00	2,00
4	1	0,00	3	3,00	2	5	1	3	3	5	3,20	2,00
4	1	0,00	4	2,00	1	4	1	5	4	5	3,80	2,00
1	1	0,00	5	5,00	4	5	1	5	5	1	4,00	2,00
3	1	0,00	4	2,00	2	1	1	5	4	5	4,00	2,00
3	1	0,00	4	4,00	1	1	4	4	3	3	3,00	3,00
1	5	0,00	5	3,00	1	1	4	4	5	5	4,00	3,00
4	1	0,00	4	2,00	1	1	1	5	5	5	4,00	2,00
3	2	0,00	4	2,00	2	4	3	5	5	4	4,00	2,00
3	1	0,00	4	2,00	1	1	1	5	5	5	4,00	3,00
2	1	0,00	4	3,00	1	1	1	5	5	5	4,00	3,00
1	1	0,00	5	5,00	5	1	2	5	5	5	5,00	3,00
1	5	0,00	5	1,00	5	5	5	5	5	4	4,80	3,00
1	4	0,00	5	5,00	3	1	1	5	5	5	4,60	1,00
3	1	0,00	3	3,00	1	1	1	1	1	1	1,40	3,00
1	1	0,00	2	2,00	4	1	1	5	2	4	3,40	2,00
4	2	0,00	3	2,00	1	2	2	5	4	5	3,60	3,00
2	1	0,00	3	1,00	4	4	3	5	3	4	3,80	2,00
2	1	0,00	5	3,00	3	3	1	5	5	5	4,60	3,00
5	1	0,00	2	1,00	1	1	1	4	2	4	2,60	2,00
5	1	0,00	1	2,00	1	1	1	4	1	1	1,60	2,00
5	1	0,00	1	1,00	1	1	1	1	1	4	1,60	3,00
1	2	0,00	1	1,00	5	1	1	3	3	5	3,40	3,00

3	1	0,00	1	1,00	4	5	2	3	5	4	3,40	3,00
1	4	0,00	4	2,00	1	4	1	4	4	4	3,40	1,00
1	2	0,00	4	3,00	3	3	3	5	5	2	3,80	3,00
4	1	0,00	2	1,00	1	4	1	4	5	4	3,20	3,00
3	4	0,00	4	1,00	4	3	4	5	5	5	4,60	1,00
2	1	0,00	2	2,00	1	3	1	5	3	4	3,00	3,00
1	1	0,00	4	2,00	1	1	1	4	4	4	3,40	1,00
4	4	0,00	4	2,00	3	5	4	4	4	5	4,00	2,00
1	3	0,00	5	4,00	4	5	3	4	2	2	3,40	2,00
1	2	0,00	4	3,00	2	1	1	5	5	4	4,00	3,00
4	1	0,00	5	1,00	5	5	5	5	5	5	5,00	2,00
1	1	0,00	4	3,00	4	4	1	5	5	4	4,40	1,00
5	1	0,00	1	2,00	4	1	1	4	3	4	3,20	2,00
5	4	0,00	1	4,00	1	5	5	3	2	5	2,40	1,00
3	1	0,00	3	2,00	4	1	1	5	4	5	4,20	2,00
3	2	0,00	2	4,00	5	2	2	4	4	5	4,00	1,00
4	1	0,00	1	1,00	1	1	1	4	2	4	2,40	2,00
5	3	0,00	4	5,00	5	3	4	4	2	5	4,00	3,00
3	2	0,00	3	2,00	2	3	1	5	5	1	3,20	3,00
3	4	0,00	1	5,00	4	4	4	3	3	1	2,40	2,00
5	1	0,00	3	2,00	1	1	1	5	5	4	3,60	2,00
4	1	0,00	2	2,00	2	1	1	5	3	4	3,20	2,00
5	1	0,00	1	1,00	3	1	4	2	1	5	2,40	1,00
5	1	0,00	1	5,00	1	1	1	2	3	1	1,60	2,00
5	1	0,00	1	5,00	1	1	1	2	3	1	1,60	2,00
3	1	0,00	1	1,00	1	1	1	5	2	2	2,20	3,00
1	1	0,00	2	1,00	1	1	1	5	5	5	3,60	2,00
2	1	0,00	3	4,00	4	1	2	4	5	4	4,00	2,00
4	1	0,00	1	2,00	1	1	1	4	4	4	2,80	1,00
4	5	0,00	4	1,00	3	3	4	4	4	5	4,00	3,00
1	4	0,00	5	5,00	5	5	4	5	5	5	5,00	2,00
2	3	0,00	2	4,00	2	2	2	2	2	2	2,00	1,00
3	2	0,00	4	2,00	3	3	2	5	5	2	3,80	2,00

2	3	0,00	2	3,00	3	3	3	2	1	3	2,20	3,00
2	4	0,00	5	2,00	5	5	1	5	4	3	4,40	1,00
1	4	0,00	3	2,00	2	3	1	2	3	1	2,20	2,00
1	2	0,00	4	5,00	2	1	1	5	3	5	3,80	3,00
5	1	0,00	1	1,00	1	3	2	3	1	4	2,00	3,00
1	3	0,00	3	1,00	1	2	1	4	2	2	2,40	3,00
5	1	0,00	2	1,00	1	2	2	3	2	1	1,80	3,00
5	5	0,00	2	1,00	4	1	3	4	2	4	3,20	1,00
2	3	0,00	2	3,00	1	1	2	4	5	1	2,60	3,00
1	1	0,00	5	5,00	5	3	3	3	5	5	4,60	2,00
1	1	0,00	5	5,00	3	1	1	4	5	4	4,20	3,00
5	1	0,00	2	4,00	1	1	2	4	2	5	2,80	1,00
2	1	0,00	5	2,00	4	1	2	1	4	4	3,60	2,00
2	3	0,00	1	5,00	5	1	1	4	2	5	3,40	2,00
1	1	0,00	4	5,00	1	1	5	5	1	5	3,20	3,00
1	1	0,00	4	1,00	3	5	5	1	5	5	3,60	3,00
3	1	0,00	2	1,00	1	1	4	4	2	4	2,60	2,00
4	1	0,00	1	1,00	1	2	2	4	1	4	2,20	3,00
5	1	0,00	4	1,00	1	4	1	5	3	5	3,60	3,00
5	3	0,00	4	1,00	4	5	3	5	3	5	4,20	3,00
1	2	0,00	5	1,00	4	5	4	5	5	5	4,80	3,00
3	1	0,00	3	1,00	1	1	1	4	1	4	2,60	2,00
4	3	0,00	2	4,00	3	4	2	4	4	5	3,60	2,00
5	1	0,00	1	2,00	3	1	4	5	1	2	2,40	3,00
5	1	0,00	2	2,00	2	1	1	2	4	4	2,80	2,00
4	1	0,00	4	2,00	1	4	4	5	5	5	4,00	3,00
5	1	0,00	1	1,00	1	1	5	1	1	1	1,00	3,00
1	3	0,00	2	1,00	5	3	5	2	2	5	3,20	2,00
2	4	0,00	1	1,00	2	1	2	2	2	1	1,60	3,00
4	3	0,00	1	1,00	3	1	5	4	1	5	2,80	2,00
1	3	0,00	5	2,00	4	5	5	5	3	3	4,00	3,00
5	1	0,00	2	2,00	1	1	5	5	5	5	3,60	3,00
5	3	0,00	3	2,00	4	3	3	2	5	2	3,20	3,00

4	1	0,00	2	1,00	1	1	1	4	4	4	3,00	3,00
3	3	0,00	3	2,00	4	3	4	4	3	4	3,60	3,00
5	1	0,00	1	2,00	1	2	1	2	1	1	1,20	2,00
4	2	0,00	5	4,00	3	3	1	5	5	4	4,40	3,00
3	1	0,00	3	2,00	1	1	1	5	4	4	3,40	3,00
1	1	0,00	1	5,00	5	1	1	5	5	5	4,20	3,00
1	1	0,00	4	3,00	4	1	1	5	4	4	4,20	2,00
3	3	0,00	3	2,00	2	5	2	3	3	3	2,80	2,00
1	3	0,00	4	1,00	4	5	1	5	3	3	3,80	3,00
4	1	0,00	2	4,00	1	1	1	5	4	2	2,80	3,00
3	5	0,00	2	4,00	2	2	2	2	3	1	2,00	3,00
2	2	0,00	2	2,00	4	2	4	1	2	5	2,80	3,00
5	1	0,00	2	3,00	4	1	1	1	1	2	2,00	2,00
3	2	0,00	2	2,00	4	5	1	5	2	2	3,00	3,00
5	1	0,00	1	1,00	2	1	1	4	1	1	1,80	3,00
2	1	0,00	3	5,00	3	2	2	5	3	5	3,80	3,00
3	5	0,00	4	3,00	1	1	1	5	5	5	4,00	2,00
2	2	0,00	3	4,00	4	3	3	5	5	5	4,40	3,00
1	5	0,00	3	3,00	3	3	3	5	3	3	3,40	3,00
1	1	0,00	3	3,00	4	1	1	3	5	5	4,00	2,00
4	2	0,00	1	1,00	2	5	1	5	4	5	3,40	3,00
1	1	0,00	2	2,00	3	1	1	5	5	5	4,00	3,00
5	3	0,00	1	1,00	1	5	3	3	2	5	2,40	3,00
1	1	0,00	2	2,00	1	1	3	1	2	3	1,80	1,00
5	1	0,00	2	2,00	4	4	4	4	4	4	3,60	2,00
4	1	0,00	1	1,00	4	1	1	4	3	1	2,60	3,00
3	1	0,00	3	5,00	1	1	1	5	5	4	3,60	3,00
2	1	0,00	1	4,00	5	1	1	4	4	5	3,80	3,00
2	1	0,00	3	3,00	3	1	1	3	3	3	3,00	3,00
1	1	0,00	1	2,00	1	1	1	5	5	5	3,40	3,00
4	1	0,00	1	1,00	1	1	1	2	2	2	1,60	2,00
5	1	0,00	3	1,00	3	1	5	4	4	5	3,80	2,00
5	1	0,00	2	1,00	1	1	5	4	4	5	3,20	2,00

1	1	0,00	3	5,00	3	4	1	5	4	4	3,80	3,00
1	1	0,00	5	1,00	1	1	1	5	5	5	4,20	3,00
5	1	0,00	2	3,00	1	3	4	5	3	5	3,20	2,00
3	1	0,00	4	5,00	4	1	1	5	5	5	4,60	3,00
5	1	0,00	3	2,00	1	4	1	4	1	2	2,20	1,00
4	2	0,00	3	2,00	4	2	5	4	3	4	3,60	3,00
5	1	0,00	3	1,00	1	1	1	3	3	5	3,00	2,00
4	4	0,00	2	4,00	2	4	2	4	4	2	2,80	3,00
3	1	0,00	3	2,00	1	1	1	4	4	2	2,80	3,00
1	1	0,00	5	5,00	4	1	1	1	5	2	3,40	2,00
5	1	0,00	1	3,00	1	1	2	1	5	5	2,60	3,00
4	1	0,00	4	1,00	1	1	1	4	3	2	2,80	1,00
5	2	0,00	1	1,00	2	1	1	1	1	5	2,00	3,00
4	1	1,00	4	2,00	1	1	1	2	2	4	2,60	3,00
3	2	2,00	2	4,00	2	1	1	2	2	2	2,00	3,00
3	1	1,00	3	3,00	3	3	3	4	3	4	3,40	3,00
5	1	1,00	1	3,00	2	2	2	4	2	3	2,40	3,00
2	1	1,00	2	4,00	3	1	1	2	2	5	2,80	3,00
1	1	1,00	3	1,00	3	2	2	5	5	3	3,80	3,00
5	1	1,00	1	1,00	4	1	1	1	1	1	1,60	3,00
4	1	1,00	3	2,00	3	3	3	4	4	3	3,40	1,00
5	1	1,00	4	2,00	1	1	1	4	4	1	2,80	3,00
5	1	1,00	1	1,00	4	2	1	5	1	5	3,20	2,00
5	1	1,00	1	4,00	1	3	1	4	4	1	2,20	2,00
5	1	1,00	1	1,00	1	1	1	1	1	4	1,60	3,00
5	1	1,00	4	1,00	4	2	1	5	5	5	4,60	2,00
4	1	1,00	3	1,00	4	3	4	5	5	5	4,40	2,00
5	1	1,00	4	1,00	1	1	1	2	4	4	3,00	3,00
5	1	1,00	1	1,00	3	1	2	1	1	4	2,00	3,00
5	1	1,00	4	2,00	1	1	1	4	4	1	2,80	3,00
2	3	3,00	3	4,00	2	4	1	3	2	3	2,60	1,00
5	1	1,00	2	3,00	3	1	1	5	3	4	3,40	3,00
5	1	1,00	3	1,00	1	1	1	5	3	5	3,40	3,00

5	1	1,00	3	2,00	4	1	1	4	4	4	3,80	2,00
5	1	1,00	2	4,00	2	2	2	2	2	2	2,00	2,00
2	3	3,00	3	5,00	2	2	3	3	2	4	2,80	1,00
5	1	1,00	1	5,00	1	1	1	2	1	1	1,20	3,00
4	2	2,00	4	4,00	1	4	1	2	1	1	1,80	2,00
5	1	1,00	1	1,00	1	1	1	4	1	4	2,20	3,00
4	4	4,00	4	3,00	3	3	4	4	3	4	3,60	2,00
5	1	1,00	1	2,00	5	1	1	2	1	5	2,80	2,00
4	2	2,00	2	2,00	4	4	4	4	4	5	3,80	1,00
3	1	1,00	2	1,00	4	1	2	5	2	4	3,40	1,00
4	1	1,00	1	2,00	2	2	1	1	1	1	1,20	1,00
1	1	1,00	5	3,00	1	5	1	5	5	4	4,00	3,00
5	1	1,00	3	2,00	1	3	4	3	3	3	2,60	1,00
5	1	1,00	2	5,00	1	1	1	4	1	1	1,80	3,00
4	2	2,00	4	4,00	4	5	2	4	4	4	4,00	1,00
5	1	1,00	4	1,00	3	5	5	4	1	4	3,20	3,00
3	5	5,00	3	3,00	3	4	4	5	5	3	3,80	3,00
5	1	1,00	4	5,00	1	4	4	3	4	3	3,00	2,00
4	1	1,00	4	2,00	4	3	3	5	4	5	4,40	1,00
5	1	1,00	2	1,00	3	3	4	4	4	3	3,20	2,00
5	1	1,00	3	2,00	1	3	1	4	4	2	2,80	2,00
5	1	1,00	1	2,00	5	1	1	1	1	1	1,80	2,00
2	1	1,00	2	2,00	1	2	3	4	2	4	2,60	3,00
5	4	4,00	2	4,00	1	3	3	3	3	3	2,40	3,00
5	1	1,00	4	2,00	3	1	1	4	1	1	2,60	2,00
3	1	1,00	5	2,00	4	3	3	5	5	5	4,80	1,00
5	1	1,00	1	1,00	1	1	1	4	1	2	1,80	3,00
5	1	1,00	2	2,00	1	1	1	3	3	3	2,40	2,00
3	1	1,00	2	2,00	1	1	1	4	3	4	2,80	3,00
4	1	1,00	1	1,00	4	1	1	1	5	4	3,00	3,00
5	1	1,00	2	3,00	3	1	1	4	3	4	3,20	1,00
1	1	1,00	5	3,00	5	5	5	5	5	1	4,20	2,00
5	1	1,00	3	3,00	2	2	2	3	1	3	2,40	3,00

5	1	1,00	2	2,00	3	4	1	4	2	4	3,00	3,00
5	1	1,00	1	1,00	1	1	1	3	1	5	2,20	2,00
5	2	2,00	3	4,00	4	4	4	5	4	3	3,80	2,00
5	1	1,00	2	1,00	5	1	4	5	4	4	4,00	3,00
5	1	1,00	1	1,00	1	4	1	5	4	4	3,00	3,00
5	1	1,00	3	3,00	3	2	2	3	3	4	3,20	2,00
5	1	1,00	3	2,00	1	1	1	4	3	3	2,80	3,00
5	1	1,00	3	1,00	4	1	1	4	4	5	4,00	2,00
5	1	1,00	4	1,00	5	1	1	4	1	2	3,20	3,00
